# Deletion of Tsc2 in Nociceptors Reduces Target Innervation, Ion Channel Expression, and Sensitivity to Heat

**DOI:** 10.1523/ENEURO.0436-17.2018

**Published:** 2018-05-03

**Authors:** Dan Carlin, Judith P. Golden, Amit Mogha, Vijay K. Samineni, Kelly R. Monk, Robert W. Gereau, Valeria Cavalli

**Affiliations:** 1 Department of Neuroscience, Washington University School of Medicine, St. Louis, MO 63110; 2 Washington University Pain Center and Department of Anesthesiology, Washington University School of Medicine, St. Louis, MO 63110; 3 Department of Developmental Biology, Washington University School of Medicine, St. Louis, MO 63110; 4 Department of Neuroscience, Hope Center for Neurological Disorders and Center of Regenerative Medicine, Washington University School of Medicine, St. Louis, MO 63110

**Keywords:** CGRP, DRG, mTOR, nociceptor, pain, Tsc2

## Abstract

The mechanistic target of rapamycin complex 1 (mTORC1) is known to regulate cellular growth pathways, and its genetic activation is sufficient to enhance regenerative axon growth following injury to the central or peripheral nervous systems. However, excess mTORC1 activation may promote innervation defects, and mTORC1 activity mediates injury-induced hypersensitivity, reducing enthusiasm for the pathway as a therapeutic target. While mTORC1 activity is required for full expression of some pain modalities, the effects of pathway activation on nociceptor phenotypes and sensory behaviors are currently unknown. To address this, we genetically activated mTORC1 in mouse peripheral sensory neurons by conditional deletion of its negative regulator Tuberous Sclerosis Complex 2 (Tsc2). Consistent with the well-known role of mTORC1 in regulating cell size, soma size and axon diameter of C-nociceptors were increased in Tsc2-deleted mice. Glabrous skin and spinal cord innervation by C-fiber neurons were also disrupted. Transcriptional profiling of nociceptors enriched by fluorescence-associated cell sorting (FACS) revealed downregulation of multiple classes of ion channels as well as reduced expression of markers for peptidergic nociceptors in Tsc2-deleted mice. In addition to these changes in innervation and gene expression, Tsc2-deleted mice exhibited reduced noxious heat sensitivity and decreased injury-induced cold hypersensitivity, but normal baseline sensitivity to cold and mechanical stimuli. Together, these data show that excess mTORC1 activity in sensory neurons produces changes in gene expression, neuron morphology and sensory behavior.

## Significance Statement

Mechanistic target of rapamycin complex 1 (mTORC1) activation promotes regeneration of injured peripheral axons, however it may have a negative effect on target innervation as well as alter normal sensory function and injury-induced pain. Acute and chronic mTORC1 inhibition may have different effects on sensory behavior. For the mTORC1 pathway to be considered as a therapeutic target to promote nerve regeneration, it is necessary to gain an understanding of the effects of chronic pathway activation on nociceptors and sensory behavior. We show that constitutive mTORC1 activation in nociceptors via conditional deletion of its negative regulator tuberous sclerosis 2 (Tsc2) results in anomalies in cell morphology and gene expression that may underlie the decrease in noxious heat sensitivity and the attenuated nerve injury-induced cold hypersensitivity observed in Tsc2-deleted mice.

## Introduction

The mechanistic target of rapamycin complex 1 (mTORC1) is a potent regulator of cellular growth affecting downstream processes such as protein translation, autophagy, and cellular metabolism (Saxton and Sabatini, 2017). mTORC1 activation has been shown to promote extensive axon regeneration following traumatic injury in both the permissive peripheral and the restrictive central nervous system environments (Park et al., 2008; Abe et al., 2010; Liu et al., 2010). Optimal functional recovery after nerve injury requires therapeutic interventions that mediate efficient target reinnervation and minimize injury-induced neuropathic pain. Increased mTORC1 activity can result in defects in target innervation. For instance, mice with mutations in the negative mTORC1 regulator tuberous sclerosis 2 (Tsc2) exhibit inappropriately targeted retinogeniculate projections or reduced innervation of glabrous skin (Abe et al., 2010; Nie et al., 2010). However, the functional consequences of this disrupted sensory target innervation are unknown.

Studies using local, systemic or intrathecal administration of mTORC1 inhibitors have shown that attenuation of mTORC1 signaling decreases inflammation- or nerve injury-induced pain and bone cancer pain (Price et al., 2007; Jiménez-Díaz et al., 2008; Geranton et al., 2009; Obara et al., 2011; Liang et al., 2013; Lutz et al., 2015; Jiang et al., 2016). A number of studies have also shown that acute local or systemic administration of mTORC1 inhibitor does not alter sensory thresholds in naïve mice or rats (Jiménez-Díaz et al., 2008; Geranton et al., 2009; Obara et al., 2011). Interestingly, chronic inhibition of mTORC1 results in mechanical allodynia in naïve mice by a mechanism that involves feedback activation of ERK signaling in sensory neurons (Melemedjian et al., 2013). These studies indicate that mTORC1 signaling modulates pain behavior in both naïve animals and in the context of injury. However, pharmacological mTORC1 inhibition may also have effects on non-neuronal cells that can impact pain behavior in addition to potentially attenuating mTORC1 signaling in central nervous system regions involved in pain transmission including spinal dorsal horn neurons and cortical neurons (Geranton et al., 2009; Asante et al., 2010; Obara et al., 2011; Beirowski et al., 2017; Kwon et al., 2017). As such, the specific role of the pathway in peripheral sensory neurons is less clear.

In peripheral sensory axons, an active phosphorylated form of mTORC1 has been shown to colocalize predominantly with markers of A-fiber axons, but was also shown to be expressed in a small percentage of C-fiber axons in both the skin and the dorsal root (Jiménez-Díaz et al., 2008; Geranton et al., 2009; Obara et al., 2011). However, mTOR protein is expressed in the soma of both classes of neurons in dorsal root ganglia (DRG; Xu et al., 2010), suggesting that mTORC1 may have functions that are specific to cell type and/or subcellular localization. Local protein synthesis in sensory axons is required for both primary and secondary hyperalgesia (Obara et al., 2012), and mTORC1 signaling is a potent activator of protein translation in sensory axons (Cho et al., 2014; Khoutorsky and Price, 2018; Terenzio et al., 2018). mTORC1 signaling has also been shown to mediate protein translation in sensory neurons in response to some pain-inducing stimuli (Melemedjian et al., 2010). While these studies have shown a requirement for mTORC1 in mediating pain responses, little is known about the consequences of excess mTORC1 activation. Mice with a genetic deletion of Eukaryotic initiation factor 4E-binding protein 1 (4EBP1), a negative regulator of protein translation that is directly inhibited by mTORC1 activity, exhibit mechanical hypersensitivity, which is reported to be spinally mediated. However, 4EBP1 was deleted in all cells thereby precluding specific analysis of peripheral sensory neurons function (Khoutorsky et al., 2015). As such, the response of sensory neurons to excess mTORC1 signaling remains to be elucidated.

In the present study, we investigated the consequences of activation of mTORC1 in sensory neurons on target innervation and sensory behavior by employing a genetic approach to delete its negative regulator Tsc2 using the sensory neuron-specific Nav1.8-Cre mouse. This strategy results in Cre expression predominantly in C-nociceptors, but also in a subpopulation of sensory neurons with myelinated axons (Agarwal et al., 2004; Shields et al., 2012). In the resulting Nav-Tsc2 mice, nociceptors displayed impaired peripheral and central target innervation as well as a disruption in nociceptor phenotype and sensory behavior-related gene expression. Naive Tsc2-deleted mice showed reduced sensitivity to noxious heat but unchanged cold and mechanical sensitivity compared to control mice. Interestingly, Tsc2-deleted mice exhibited decreased nerve injury-induced cold hypersensitivity; however, it is possible that deficiencies in innervation and gene expression may account for this phenotype.

## Materials and Methods

### Animals

*Tsc2^fl/fl^* (floxed allele; RRID:MGI:3712786; Hernandez et al., 2007), *Tsc2^null/+^* (targeted null allele; RRID:MGI:2174787; Onda et al., 1999), *Nav1.8^Cre/+^* (MGI:3042874; Agarwal et al., 2004), and *Rosa26-ZsGreen* (RRID:IMSR_JAX:007906; Madisen et al., 2010) mice were described previously. To generate experimental animals, *Nav1.8^Cre/+^; Tsc2^null/+^*mice were crossed with *Tsc2^fl/fl^* mice. *Nav1.8^Cre/+^; Tsc2^null/fl^*mice are referred to as Nav-Tsc2 mice. Littermate animals with genotypes *Tsc2^fl/+^*, *Tsc2^null/fl^*, and *Nav1.8^Cre/+^; Tsc2^fl/+^* were used as controls as they showed no phenotypic differences from each other. For experiments using the *Rosa26-ZsGreen* reporter, control; Rosa-GFP refers specifically to *Nav1.8^Cre/+^; Tsc2^fl/+^*; *Rosa26-ZsGreen^GFP/+^* while Nav-Tsc2; Rosa-GFP refers to *Nav1.8^Cre/+^; Tsc2^fl/null^*; *Rosa26-ZsGreen^GFP/+^.* Genotype was determined by PCR at weaning. Male and female mice aged 7–18 weeks were used for experiments unless noted otherwise. All animal procedures were performed in accordance with the Washington University School of Medicine animal care committee’s regulations.

### Western blotting

Adult L4 DRG were dissected into cell lysis buffer (Cell Signaling) with protease and phosphatase inhibitors (Roche Applied Sciences) and manually homogenized. Protein concentration was determined by DC protein assay (Bio-Rad Laboratories) against bovine serum albumin standards. 10 μg total protein was loaded onto 10% polyacrylamide gels. Membranes were blotted with antibodies directed against the following proteins: α-tubulin (1:20 000; Abcam catalog #ab18251, RRID:AB_2210057), S6 kinase (1:1000; Cell Signaling Technology catalog #2708, RRID:AB_390722), phospho-S6 kinase T389 (1:750; Cell Signaling Technology catalog #9234, RRID:AB_2269803), Tsc2 (1:1000; Cell Signaling Technology catalog #4308), and rabbit IgG conjugated to horseradish peroxidase (1:10,000; Thermo Fisher catalog #656120). Initially blots were probed for Tsc2 and phospho-S6 kinase, then membranes were stripped in 60 mM Tris-HCl, 2% sodium dodecyl sulfonate, pH 6.8 at 50°C for 30 min, washed extensively, and probed in succession for S6 kinase and α-tubulin. Blots were developed with SuperSignal West Dura (ThermoFisher) and imaged with a ChemiDoc MP imaging system (Bio-Rad Laboratories).

### Immunohistochemistry

Isolated footpads were fixed by immersion in 2% paraformaldehyde, 15% saturated picric acid in PBS. Spinal cord and DRG were fixed via transcardial perfusion with PBS followed by 4% paraformaldehyde, isolated and immersed in 4% paraformaldehyde. Following several washes, tissue was cryoprotected in 30% sucrose in PBS and sectioned using a cryostat set to cut 18-, 20-, or 30-μm sections for DRG, spinal cord and footpad, respectively.

Immunostaining was performed as follows. Following a brief post-fixation in 4% paraformaldehyde and several washes in PBS with 0.1% Triton X-100 (PBSTx), sections were blocked using 5% donkey serum dissolved in PBSTx. Subsequently, sections were incubated overnight at 4°C in the following primary antibodies diluted in blocking reagent: chicken anti-βIII tubulin (1:500 for footpad; Abcam catalog #ab107216, RRID:AB_10899689), rabbit anti-βIII tubulin (1:500 for spinal cord, DRG; BioLegend catalog #802001, RRID:AB_291637), goat anti-CGRP (1:400; Bio-Rad/AbD Serotec catalog #1720-9007, RRID:AB_2290729), rabbit anti-TrkA (1:300; Millipore catalog #06-574, RRID:AB_310180), guinea pig anti-substance P (SP; 1: 250; Abcam catalog #ab10353, RRID:AB_297089). *Griffonia simplicifolia* isolectin B4 (IB4) directly conjugated to Alexa Fluor 488 or Alexa Fluor 594 (1:250; Thermo Fisher catalog #I21411 and I21413) was incubated with primary antibodies. Mouse anti-Neurofilament 200 antibody (NF200; Sigma-Aldrich catalog #MAB5266) was directly conjugated to Alexa Fluor 488 or Alexa Fluor 594 using Apex labeling kit (Thermo Fisher) and incubated with primary antibodies at 1:200 dilution. Tissue was washed several times with PBSTx, incubated with fluorescent-conjugated secondary antibodies (1:500; Thermo Fisher Scientific) and DAPI (1:1000) diluted in blocking reagent, washed, and mounted in ProLong Gold antifade mountant (Thermo Fisher Scientific). Images were taken with a Nikon A1R confocal or TE-2000E compound microscope and analyzed in ImageJ or FIJI (NIH).

### TMP histochemistry

Following two washes with 40 mM Trizma-Maleate (TM) buffer, pH 5.6, adult L4 DRG or spinal cord sections were washed with TM buffer containing 8% (w/v) sucrose. Samples were then incubated at 37°C for two hours in TM buffer containing 8% sucrose, 6 mM thiamine monophosphate chloride and 2.4 mM lead nitrate. Samples were washed once with 2% acetic acid for one minute, then washed three times with TM buffer and developed for 10 s with an aqueous solution of 1% sodium sulfide. The reaction was quenched with TM buffer. Samples were mounted in ProLong Gold mountant (Thermo Fisher) and imaged by differential interference contrast (DIC) microscopy. Five sections per animal separated by ≥54 μm (DRG) or ≥180 μm (spinal cord) were analyzed.

### Electron microscopy

Sciatic nerves from adult (8–18 weeks old) and postnatal day 29 (P29) animals were isolated and immersed in 2% glutaraldehyde, 4% paraformaldehyde in sodium cacodylate overnight at 4°C. Nerves were again fixed in 2% osmium tetroxide in sodium cacodylate for 1 h and then treated with gradually increasing concentration of ethanol (25%, 50%, 70%, 80%, 95%, 100% v/v) for 20 min each, followed by propylene oxide for 20 min. Nerves were treated with propylene oxide:EPON mix (2:1 for 1 h and then 1:1 overnight) followed by embedding in 100% EPON. Embedded nerve samples were baked at 65°C for 2–3 d. Solidified samples were cut at 70 nm on an ultra-microtome. Sections were stained with 8% uranyl acetate followed by Sato’s lead stain before image acquisition on a Jeol (JEM-1400) electron microscope. Images were recorded with an Advanced Microscopy Techniques V601 digital camera.

### Image analysis

#### DRG neuron counting

The total number of L4 DRG neurons was quantified by counting βIII tubulin-positive profiles (TuJ1). DRG neuron subtypes were counted as TuJ1-positive cells that colabeled with subtype-specific markers. All positive profiles that were also DAPI-positive were counted on every fourth section and the result multiplied by four to obtain total L4 counts for DRG neurons and subtypes.

#### DRG cell size

Using the polygon tool in FIJI software, TMPase-positive and CGRP-positive cells were outlined. Area measurements were recorded for up to 20 TMPase-positive or CGRP-positive/NF200-negative or 10 CGRP-positive/NF200-positive neurons from a random region of each section and repeated until 100 TMPase-positive or CGRP-positve/NF200-negative or 50 CGRP-positive/NF200-positive neurons were analyzed for each animal. Average cell area for each animal was determined. In addition, the number of cells of small, medium, and large diameter were determined. Cells with area <314 μm^2^, corresponding to a spherical diameter of <20 μm^2^, were characterized as small diameter. Cells with area 315–706 μm^2^, corresponding to a spherical diameter of 20–30 μm^2^, were characterized as medium diameter. Cells with area >707 μm^2^, corresponding to a spherical diameter of >30 μm^2^, were characterized as large diameter.

#### Glabrous skin (footpad)

TuJ1-positive fibers entering the epidermis were counted by focusing up and down through glabrous skin sections from the two most distal footpads of the hind paw. A stitched single plane image was then taken of the region of interest (ROI) and the length of the epidermal-dermal border was traced. Total fibers and epidermal border length of both footpads were determined for each section. Results from five sections per animal separated by ≥120 μm were averaged. Representative sections were imaged as a z-series and a max intensity projection was generated in ImageJ.

#### Nav-Tsc2;Rosa-GFP spinal cord

Lumbar spinal cord sections were stained for IB4 conjugated to Alexa Fluor 594 and imaged by confocal microscopy with 2X average line scans and sequential scanning using a 60× objective. To determine GFP density, GFP signal was made binary through default auto-thresholding in ImageJ. The location of IB4 labeling in the superficial dorsal horn did not appear to be altered by Tsc2 deletion. We therefore used IB4 staining as a marker for Lamina II. Lamina I was defined as the region within the dorsal horn that was dorsal to IB4 labeling. Laminas I and II were outlined, and the percentage GFP-positive area was determined for each. Five sections per animal separated by ≥180 μm were analyzed and averaged. The average GFP density for each sample was normalized to the percentage of GFP-positive neurons in the L4 DRG of the same animals. These values were then normalized to the mean for controls.

#### Sciatic nerve electron microscopy

Axon diameter measurements were made on images of 2000× magnification by averaging the lengths of two lines through the center of the axon at right angles. At least 20 Remak bundles were analyzed per animal, corresponding to >200 axons for each P29 animal and >125 axons for each adult animal.

### DRG neuron dissociation and flow cytometry

L4 DRG from control; Rosa-GFP and Nav-Tsc2; Rosa-GFP mice contralateral to a sciatic nerve crush were isolated 3 d after injury in Hanks’ balanced salt solution with 10 mM HEPES (HBSS-H). Ipsilateral DRG were not analyzed in this study. DRG were treated at 37°C with consecutive applications of papain (15 U/ml) and collagenase (1.5 mg/ml) in HBSS-H. After washes, DRG were dissociated by trituration, passed through a 70-μm cell strainer, resuspended in PBS with 2% fetal calf serum and subjected to flow cytometry. Cells were run through an 85-μm nozzle at 45 psi. on a BD FACS Aria II machine and positively sorted for GFP signal.

### Quantitative PCR (qPCR)

For whole DRG, adult L4 DRG from control mice were isolated bilaterally, lysed, and homogenized. For fluorescence-associated cell sorting (FACS)-sorted cells, adult L1-L6 lumbar DRG from control; Rosa-GFP mice were isolated, dissociated and sorted by flow cytometry for GFP signal. For each sample, 2500 GFP-positive cells were sorted into lysis buffer from PureLink RNA Mini kit with 5% RiboLock RNase Inhibitor (Thermo Fisher). Total RNA was extracted with PureLink RNA Mini kit according to manufacturer’s instructions (Thermo Fisher Scientific). RNA concentration was determined by NanoDrop 2000 (Thermo Fisher Scientific) for whole DRG. All samples were reverse transcribed with High Capacity cDNA Reverse Transcription kit (Applied Biosystems). qPCR was performed with PowerUp SYBR Green master mix on 2-ng cDNA from whole DRG or cDNA from 62.5 FACS-sorted cells with a QuantStudio 6 Flex and analyzed with QuantStudio Real-Time PCR Software v1.3 (Applied Biosystems). Validated primer sequences were obtained from PrimerBank where available (Wang et al., 2012). Additional primers were designed and validated for amplification efficiency standard curve analysis. Single amplified products were noted from melting point analyses and agarose gel electrophoresis. All primer sequences are found in Table 1. ΔΔCt analysis was used to normalize target gene expression data to the geometric mean of Ribosomal protein L13a (*Rpl13a*) and *Gapdh* reference gene expression. Target gene expression from FACS-sorted cells was then normalized to expression from whole DRG.

**Table 1.** Primer sequences used for qPCR analysis

Gene name	Forward primer (5'-3')	Reverse primer (5'-3')
*Ntrk1 / TrkA*	GCCTAACCATCGTGAAGAGTG	CCAACGCATTGGAGGACAGAT
*Scn10a / Nav1.8*	TCCGTGGGAACTACCAACTTC	GCTCGCCATAGAACCTGGG
*Ntrk2 / TrkB*	CTGGGGCTTATGCCTGCTG	AGGCTCAGTACACCAAATCCTA
*Ntrk3 / TrkC*	CCGCATCCCAGTCATTGAGAA	TGACCTTGGGTAAGACACATCC
*Periaxin*	CTCAGCTTGCAAGAAGGGGA	CGTACCAGCTTGGCCACTTT
*Egr2 / Krox20*	GGCTCAGTTCAACCCCTCTC	GCGCAAAAGTCCTGTGTGTT

### RNA-seq analysis

100 L4 DRG cells were FACS-sorted for GFP into 10-μl Clontech lysis buffer with 5% RiboLock RNase Inhibitor for each sample. Three technical replicates of 100 cells each were sorted, and libraries were prepped and sequenced separately for each biological replicate. All samples were submitted to the Genome Technology Access Center at Washington University in St Louis for library preparation and sequencing. Library preparation was performed using the SMARTer Ultra Low RNA kit for Illumina Sequencing (Clontech) per manufacturer’s protocol. cDNA was amplified for 13 cycles and then fragmented using a Covaris E220 sonicator using peak incident power 18, duty cycle 20%, cycles/burst 50, time 120 s at room temperature. cDNA was blunt ended, had an A base added to the 3’ends, and then had Illumina sequencing adapters ligated to the ends. Ligated fragments were then amplified for 15 cycles using primers incorporating unique index tags. Fragments were sequenced on an Illumina HiSeq-3000 using single reads extending 50 bases. Samples were QC’d using FastQC, aligned to mm10 using STAR-align, and counted using HTseq-count. Technical replicates were collapsed in RStudio and differential expression determined using DESeq2. Adjusted *p* < 0.05 and log2 fold change >0.5 or <-0.5 were used as the cutoff for differential expression. Significantly regulated genes were uploaded to MetaCore for downstream analysis to determine Gene Ontology (GO) processes and molecular functions that were significantly altered in Nav-Tsc2 mice.

### Data deposition

RNA-seq FastQ files were deposited at the NCBI GEO database (http://www.ncbi.nlm.nih.gov) under accession number GSE112499.

### Behavioral analysis

#### Accelerating rotarod

Mice were assessed for gross motor function using an accelerating Rotarod (Ugo Basile). Mice were trained until they were able to remain on the Rotarod (4 rpm) for 120 s. 1 h after training, five consecutive trials were performed on an accelerating Rotarod with 5 min between trials. Latency to fall was measured as the apparatus accelerated from 4–40 rpm over 5 min.

#### Open field activity

Locomotion was assessed using an open field (42 × 42 × 30 cm, length × width × height) equipped with a Versamax Animal Activity Monitoring System (AccuScan Instruments). Before testing, mice were habituated to the room in their home cages for at least 1 h. Mice were then placed in the open field during individual trials and allowed to freely explore after the experimenter exited the room. The horizontal activity, distance traveled, and time moving during the 1 h trial were determined by the Versamax software.

#### Pole climb down

The pole test was used to evaluate performance in a complex motor task. Mice are placed on a vertical metal pole that is 49 cm in height and 0.9 cm in diameter with the head of the mouse oriented upward. The time required for the mouse to turn around such that the mouse’s head is oriented downward and the hind limbs are straddling the pole is recorded. In addition, the time required for the mouse to climb down to the base of the pole is recorded.

#### von Frey test

Varying diameter von Frey monofilaments (Stoelting) were pressed against the plantar surface of the hind paw until the filament bent. The force applied to the hind paw is dependent on the diameter of the filament. The up/down method described by Chaplan was used to determine the mechanical withdrawal threshold (Chaplan et al., 1994). Three trials were performed on each paw. The three trials were averaged to obtain the withdrawal threshold for each paw.

#### Cold plantar test

Mice were tested for cold sensitivity in a manner described previously (Brenner et al., 2012). Briefly, mice were acclimated on a glass plate. Crushed dry ice pellet was pressed against the glass underneath the hindpaw. Withdrawal latency was measured, with withdrawal defined as any action to move the paw vertically or horizontally away from the cold glass. An interval of 7 min was allowed between paws and 15 min between trials. The average latency times for two trials from both hindpaws were averaged for each mouse.

#### Acetone test

Sensitivity to a cold stimulus was measured using the acetone test. One drop of acetone was applied to the plantar surface of the hind paw using a 1 ml-syringe. Mice were observed for 5 min after each acetone application. The amount of time spent in spontaneous pain behavior was recorded. Spontaneous pain behavior was defined as shaking, flinching, or licking of the paw as well as holding the paw in an elevated position. Five trials were performed on each hind paw, and the response times for each of the five trials were summed to determine the response time for each paw.

#### Hargreaves test

The thermal threshold was determined by measuring the withdrawal latency to a radiant heat source (ITTC Instruments) applied to the plantar surface of the hindpaw in three separate trials for each hindpaw with a 15-min interval between trials. The withdrawal threshold was determined by averaging the withdrawal latency obtained in each of the three trials. The thermal threshold was determined at an active intensity of 18 (AI18).

#### Chronic constriction injury (CCI)

Under 2.5% isoflurane anesthesia, the sciatic nerve was loosely ligated with 6-0 chromic gut sutures. Two ligatures separated by 3–5 mm were placed around the sciatic nerve. The acetone test was performed before surgery and at specified times after surgery on contralateral and ipsilateral hind paws.

### Statistical analysis

Experimenters performing surgery, behavioral observations or image analysis were blinded to genotype. Statistically significant differences were determined by two-tailed unpaired *t* test, two-way RM ANOVA, or Sidak’s multiple comparison test using GraphPad Prism software. *F* and *p* values are reported in text for ANOVA results while only *p* values are reported for *t* tests. Sidak’s multiple comparison test was used on individual data points for figures where two-way ANOVA was reported in text. All statistical data are included in Table 2. Statistical significance was defined by *p* < 0.05.

**Table 2.** Statistical table

Figure	Statistical test	*N*	Statistical significance
1*E*	Unpaired *t* test, two-tailed	TMPase: *N* = 6	TMPase: *p* < 0.0001
Cell area		CGRP: *N* = 5	CGRP+,NF200-: *p* < 0.0001
			CGRP+,NF200+: *p* = 0.4803
Table 3	Unpaired *t* test, two-tailed	*N* = 5–6	See Table 3
Cell distribution			
2B	Unpaired *t* test, two-tailed	Adult: *N* = 6	Adult control vs adult Nav-Tsc2: *p* = 0.0047
Axon diameter		P29: *N* = 5	P29 control vs P29 Nav-Tsc2: *p* = 0.0442
			Adult control vs P29 control: *p* = 0.318
			Adult Nav-Tsc2 vs P29 Nav-Tsc2: *p* = 0.0258
2C	Unpaired *t* test, two-tailed	Adult: *N* = 6	Adult control vs adult Nav-Tsc2: *p* = 0.0055
Axons >1 micron		P29: *N* = 5	P29 control vs P29 Nav-Tsc2: *p* = 0.2076
			Adult control vs P29 control: *p* = 0.4211
			Adult Nav-Tsc2 vs P29 Nav-Tsc2: *p* = 0.0125
2D	Unpaired *t* test, two-tailed	Adult: *N* = 6	Adult control vs adult Nav-Tsc2: *p* = 0.0371
Axons/bundle		P29: *N* = 5	P29 control vs P29 Nav-Tsc2: *p* = 0.0747
			Adult control vs P29 control: *p* = 0.5018
			Adult Nav-Tsc2 vs P29 Nav-Tsc2: *p* = 0.0202
3C	Unpaired *t* test, two-tailed	*N* = 5	TuJ1: *p* < 0.0001
Skin innervation			
3F	Unpaired *t* test, two-tailed	*N* = 5	Lamina I: *p* = 0.0403
GFP density			Lamina II: *p* = 0.0002
5C,F,G,L	Unpaired *t* test, two-tailed	*N* = 5	Total neurons: *p* = 0.081
DRG neuron		SP: *N* = 6	NF200 total: *p* = 0.314
counting			SP total: *p* = 0.0006
			CGRP+,NF200-: *p* < 0.0001
			CGRP+,NF200+: *p* = 0.0007
			TrkA+,NF200-: *p* < 0.0001
			TrkA+,NF200+: *p* = 0.0005
			IB4 total: *p* = 0.0001
			IB4+,NF200+: *p* = 0.0037
6C	Unpaired *t* test, two-tailed	*N* = 4 whole DRG	Nav1.8: *p* = 0.0613
qPCR of		*N* = 3 FACS-sorted samples	TrkA: *p* = 0.0015
FACS-sorting			TrkB: *p* = 0.9758
			TrkC: *p* = 0.0020
			Prx: *p* = 0.1784
			Egr2: *p* = 0.5834
8A	Unpaired *t* test, two-tailed	Female: *N* = 13	Female: *p* = 0.2513
von Frey		Male: *N* = 12	Male: *p* = 0.462
8B	Unpaired *t* test, two-tailed	Female: *N* = 6 control, *N* = 4 cKO	Female: *p* = 0.2390
Cold plantar		male: *N* = 11 control, *N* = 10 cKO	Male: *p* = 0.2102
8C	Unpaired *t* test, two-tailed	Female: *N* = 13	Female: *p* = 0.0046
Hargreaves		Male: *N* = 9	Male: *p* = 0.0337
8D	Two-way RM ANOVA	Control: *N* = 9	Interaction: *F*_(5,75)_ = 1.82; *p* = 0.1191
Female CCI		Nav-Tsc2: *N* = 8	Time: *F*_(5,75)_ = 10.04; *p* < 0.0001
			Genotype: *F*_(1,15)_ = 9.032; *p* = 0.0089
8D	Sidak’s multiple	Control: *N* = 9	BL: adjusted *p* > 0.9999
Female CCI	comparison test	Nav-Tsc2: *N* = 8	POD 4: adjusted *p* = 0.269
			POD 6: adjusted *p* = 0.9896
			POD 20: adjusted *p* = 0.9209
			POD 27: adjusted *p* = 0.0071
			POD 34: adjusted *p* = 0.0836
8E	Two-way RM ANOVA	Control: *N* = 11	Interaction: *F*_(5,75)_ = 0.7719; *p* = 0.5723
Male CCI		Nav-Tsc2: *N* = 10	Time: *F*_(5,95)_ = 4.873; *p* = 0.0005
			Genotype: *F*_(1,19)_ = 3.403; *p* = 0.0807
8E	Sidak’s multiple	Control: *N* = 11	BL: adjusted *p* > 0.9999
Male CCI	comparison test	Nav-Tsc2: *N* = 10	POD 4: adjusted *p* = 0.9992
			POD 6: adjusted *p* = 0.9998
			POD 20: adjusted *p* = 0.3652
			POD 27: adjusted *p* = 0.236
			POD 34: adjusted *p* = 0.8045
8F	Two-way RM ANOVA	Control: *N* = 17	Interaction: *F*_(4,128)_ = 1.03; *p* = 0.3946
Rotarod		Nav-Tsc2: *N* = 17	Trial number: *F*_(4,128)_ = 60.26; *p* < 0.0001
			Genotype: *F*_(1,32)_ = 4.08; *p* = 0.0518
8F	Sidak’s multiple	Control: *N* = 17	Trial 1: adjusted *p* = 0.8576
Rotarod	comparison test	Nav-Tsc2: N = 17	Trial 2: adjusted *p* = 0.8396
			Trial 3: adjusted *p* = 0.2686
			Trial 4: adjusted *p* = 0.0812
			Trial 5: adjusted *p* = 0.1971
8G	Unpaired *t* test, two-tailed	Control: *N* = 23	*p* = 0.8027
Open field activity		Nav-Tsc2: *N* = 23	
8H	Two-way RM ANOVA	Control: *N* = 23	Interaction: *F*_(1,43)_ = 2.62; *p* = 0.1129
Pole climb down		Nav-Tsc2: *N* = 22	Trial number: *F*_(1,43)_ = 4.516; *p* = 0.0394
			Genotype: *F*_(1,43)_ = 0.04254; *p* = 0.8376
8H	Sidak’s multiple	Control: *N* = 23	Trial 1: adjusted *p* = 0.84
Pole climb down	comparison test	Nav-Tsc2: *N* = 22	Trial 2: adjusted *p* = 0.606

## Results

### Tsc2 deletion increases cell body and axon diameters of C-fiber neurons

**Figure 1.**
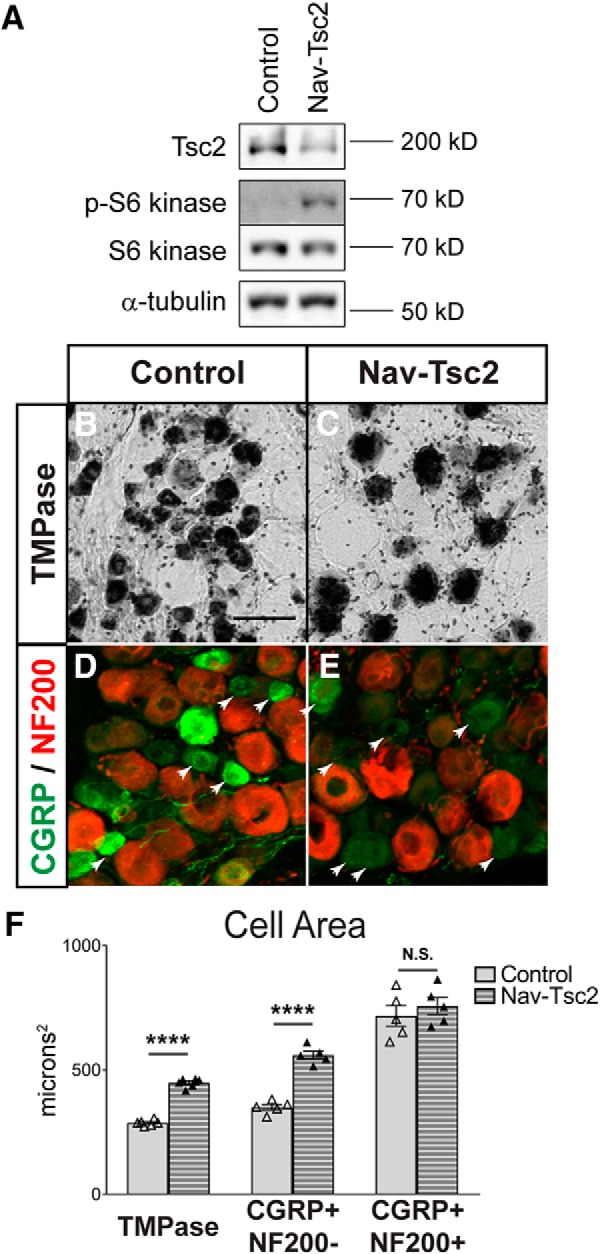
Tsc2 deletion in Nav1.8-positive neurons activates mTORC1 signaling and increases soma size. ***A***, Western blotting of L4 DRG from adult Nav-Tsc2 mice showed decreased expression of Tsc2 and increased phosphorylation of the direct mTORC1 target S6 kinase T389 relative to control mice; *N* = 8–10. ***B***, ***C***, TMP histochemistry of adult control and Nav-Tsc2 L4 DRG. Scale bar: 50 µm; *N* = 6. ***D***, ***E***, Immunohistochemistry of adult control and Nav-Tsc2 L4 DRG for CGRP and NF200. Arrows point to CGRP-positive, NF200-negative neurons; *N* = 5. ***F***, Average cell area of labeled neurons. Individual animals plotted with mean ± SEM; N.S, *p* > 0.05, ****p < 0.0001.

Previous studies have analyzed the requirement of mTORC1 for full expression of pain states primarily using pharmacological approaches to inhibit mTORC1 signaling (Jiménez-Díaz et al., 2008; Geranton et al., 2009; Ferrari et al., 2013; Melemedjian et al., 2013; Obara et al., 2011, 2015). However, the effect of chronic mTORC1 activation in peripheral neurons on sensory behavior has not been determined. To analyze the effect of chronic mTORC1 activation in peripheral sensory neurons, we generated mice with Tsc2 deletion in a subset of DRG neurons, those expressing the voltage-gated sodium channel Nav1.8, which we designate as Nav-Tsc2 mice. Cre-mediated recombination occurs in these mice after embryonic day 17.5, and in a population that comprises >90% of nociceptors and ∼40% of myelinated DRG neurons. (Agarwal et al., 2004; Shields et al., 2012). Animals with genotypes *Tsc2^fl/+^*, *Tsc2^null/fl^*, and *Nav1.8^Cre/+^; Tsc2^fl/+^* were pooled as a control group as they did not exhibit phenotypic differences from each other. Phosphorylation of p70 S6 kinase at threonine 389, a direct target of mTORC1 kinase (Isotani et al., 1999), was increased in L4 DRG of adult Nav-Tsc2 mice compared to controls (Fig. 1*A*), confirming mTORC1 activity was increased as a result of Tsc2 deletion in Nav1.8-positive DRG neurons. Residual Tsc2 protein expression in Nav-Tsc2 DRG was likely due to non-neuronal and Nav1.8-negative neuronal contributions.

As the Tsc2/mTORC1 signaling axis affects cellular metabolism by regulation of numerous growth-related processes (Saxton and Sabatini, 2017), we analyzed nociceptor cell size to validate that Tsc2 deletion has functional consequences for those neurons. Thiamine monophosphatase (TMPase) is a marker predominantly for nonpeptidergic neurons and is known to colocalize extensively with IB4 in DRG neurons (Zylka et al., 2008). We observed a 56% increase in the average cell area of TMPase-positive nonpeptidergic L4 DRG neurons from Nav-Tsc2 mice compared with control mice (*p* < 0.0001; Fig. 1*B*,*C*,*F*). This increase in average cell size was accompanied by a shift in the distribution of small, medium, and large diameter nonpeptidergic neurons. In control animals, TMPase-positive neurons were predominantly small diameter with some medium and no large diameter cells. In Nav-Tsc2 mice, some small diameter neurons were present, however most neurons were medium diameter with some large diameter neurons as well (Table 3; for cell size categories, see Materials and Methods). Therefore, Tsc2 deletion increases cell size of nonpeptidergic nociceptors.

**Table 3.** Percentage of small, medium, and large diameter neurons labeled with cell type-specific markers

	Small	Medium	Large
TMPase			
Control	67 ± 1.98%	33 ± 1.98%	0 ± 0%
Nav-Tsc2	20.83 ± 1.68%	73.83 ± 1.66%	5.33 ± 1.31%
*p* value (*N*)	<0.0001 (6)	<0.0001 (6)	0.0712 (6)
CGRP+, NF200-			
Control	41 ± 4.63%	58.4 ± 4.5%	0.6 ± 0.4%
Nav-Tsc2	4.2 ± 1.88%	77.8 ± 0.97%	18 ± 2.35%
*p* value (*N*)	<0.0001 (5)	0.0003 (5)	0.001 (5)
CGRP+, NF200+			
Control	4.4 ± 0.75%	46.8 ± 6.28%	48.8 ± 6.22%
Nav-Tsc2	2 ± 1.1%	50.8 ± 3.83%	47.2 ± 4.59%
*p* value (*N*)	0.9736 (5)	0.893 (5)	0.9918 (5)

Data are presented as mean percentage ± SEM for control and Nav-Tsc2 mice with corresponding *p* value and *N*. Small, medium, and large diameter categorized defined in Materials and Methods.

Similarly, we analyzed the cell size of peptidergic nociceptors that are CGRP-positive. This population is heterogeneous in cell size as it is comprised of both A- and C-nociceptors, which we distinguished by the presence or absence of NF200 expression, respectively. CGRP-positive neurons exhibited decreased intensity of immunostaining in Nav-Tsc2 mice, but neurons were still identifiable. The average cell area of NF200-negative peptidergic C-nociceptors in Nav-Tsc2 was increased by 60% compared to neurons from control mice (*p* < 0.0001; Fig. 1*D–F*, arrows). Additionally, these neurons exhibited a remarkably similar shift in size distribution as nonpeptidergic nociceptors whereby the percentage of medium and large diameter neurons was increased in Nav-Tsc2 mice compared with control mice (Table 3), suggesting both classes of C-nociceptors are susceptible to metabolic anomalies as a result of Tsc2 deletion. Interestingly, we did not observe a change in the average size of peptidergic A-nociceptors (CGRP, NF200 double positive) nor in the distribution of cell sizes among this population (*p* = 0.4803; Fig. 1*D–F*; Table 3), suggesting that Tsc2 deletion may have a limited effect on the regulation of soma size and/or cellular metabolism of these cells. Notably, Nav1.8-Cre is known to be expressed in >90% of CGRP-positive neurons, and is not restricted to C-nociceptors (Shields et al., 2012). The increased cell size of C-nociceptors in DRG from Nav-Tsc2 mice validates that Tsc2 deletion in these cells has functional consequences that are consistent with constitutive mTORC1 activation.

**Figure 2.**
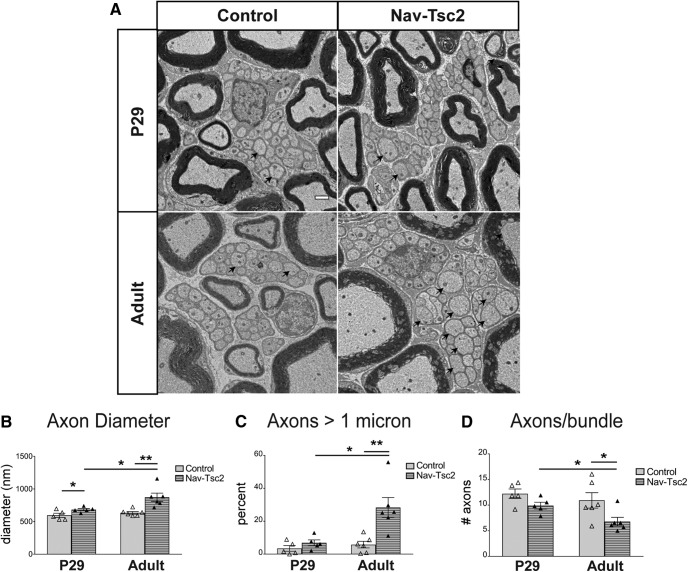
C-fiber axon diameter is increased in Nav-Tsc2 mice. ***A***, Transmission electron micrographs of representative transverse sections of sciatic nerve of P29 (*N* = 5) and adult (*N* = 6) mice. Arrows point to axons greater than one micron in diameter. Scale bar: 1 µm. ***B***, Average diameter of axons bundled by Remak Schwann cells. ***C***, Percentages of axons bundled by Remak Schwann cells that have diameter greater that one micron. ***D***, Average number of axons within individual Remak bundles. Individual animals plotted with mean ± SEM; *p < 0.05, **p < 0.01.

As cell size of C-fiber neurons was increased in Nav-Tsc2 mice, we investigated whether axon diameter was also affected. Axon diameter affects conduction velocity and alterations may influence behavioral responses to sensory stimuli. Both nonpeptidergic and peptidergic C-fiber axons are unmyelinated and associate with Remak Schwann cells in bundles within peripheral nerves (Murinson et al., 2005). To determine whether axon diameter of C-fiber neurons was affected by Tsc2 deletion, we analyzed the diameter of axons in Remak bundles of the sciatic nerve by transmission electron microscopy. The average diameter of Remak-bundled axons of adult Nav-Tsc2 mice was increased by 38% compared to controls (*p* = 0.0047; Fig. 2*A*,*B*), showing that both axon and cell body size are affected by Tsc2 deletion. Generally, axons greater than one micron in diameter become myelinated during early postnatal development, while axons less than one micron in diameter are sorted into Remak bundles. However, in adult Nav-Tsc2 mice we noted a significant increase in Remak-bundled axons greater than one micron in diameter compared to controls (*p* = 0.0055; Fig. 2*A*,*C*). In addition, we noted a decrease in the number of axons per Remak bundle in Nav-Tsc2 mice (*p* = 0.0371; Fig. 2*A*,*D*), suggesting that axon diameter may affect Remak bundle organization.

Peripheral axons are radially sorted during the first weeks of postnatal life in mouse to associate with myelinating Schwann cells (>1 μm axon diameter) or with Remak Schwann cells (<1 μm axon diameter). To determine whether the increased axon diameter in Nav-Tsc2 mice is the result of disrupted radial sorting or excess neuronal growth post-sorting, we analyzed sciatic nerves from control and Nav-Tsc2 mice at P29, after completion of radial sorting (Feltri et al., 2016). At P29, average axon diameter was already increased in Nav-Tsc2 mice compared to control mice (*p* = 0.0442; Fig. 2*A*,*B*). However, while control mice showed no age-dependent increase in average axon diameter of unmyelinated axons, average axon diameter of Nav-Tsc2 mice increased from P29 to adult (control: *p* = 0.318; Nav-Tsc2 *p* = 0.0258; Fig. 2*A*,*B*). Despite the increase in average axon diameter of Nav-Tsc2 mice at P29, the percentage of bundled axons greater than one micron in diameter was not affected (*p* = 0.2076; Fig. 2*A*,*C*). Consistently, there was no change in the number of axons per bundle at P29 in Nav-Tsc2 mice compared to control mice (*p* = 0.0747; Fig. 2*D*). However, there was a statistically significant age-related decline in the number of axons per bundle in sciatic nerves from Nav-Tsc2 mice that was not seen in controls (control: *p* = 0.5018; Nav-Tsc2 *p* = 0.0202; Fig. 2*A*,*D*). Together these data are consistent with the notion that axons are sorted properly by size during early postnatal development, and axonal hypertrophy after sorting reduces the number of axons per bundle. This may be due to Remak Schwann cell hyperplasia or Remak fragmentation or elaboration of Schwann cell processes, as has recently been described following injury (Gomez-Sanchez et al., 2017).

### Peripheral and central target innervation are preferentially disrupted by loss of Tsc2

**Figure 3.**
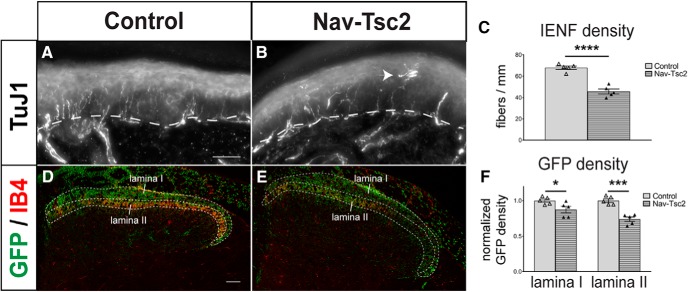
Fiber innervation is reduced in both central and peripheral targets of DRG neurons in Nav-Tsc2 mice. ***A***, ***B***, Projection of a z-stack of hindpaw glabrous skin labeled with TuJ1. Arrowhead shows aberrant fiber morphology in Nav-Tsc2 mice. Dotted line denotes epidermal-dermal border. Scale bar: 50 µm. ***C***, Number of TuJ1-positive fibers crossing the epidermal-dermal border were counted in control and Nav-Tsc2 mice; *N* = 5. ***D***, ***E***, Confocal micrographs of Rosa-GFP reporter in control and Nav-Tsc2 lumbar spinal cord transverse sections labeled with IB4. Laminas I and II used for quantification are outlined. Scale bar: 50 µm. ***F***, Normalized area fraction of GFP signal in Laminas I and II; *N* = 5. Individual animals plotted with mean ± SEM; *p < 0.05, ***p < 0.001, ****p < 0.0001.

As axon morphology was affected in Nav-Tsc2 mice and previous studies have shown that Tsc2 deletion mediated by Advillin-Cre reduces innervation of glabrous skin (Abe et al., 2010), we analyzed C-fiber target innervation in Nav-Tsc2 mice. To determine whether skin innervation is disrupted in Nav-Tsc2 mice, we performed immunohistochemistry for the pan-neuronal marker βIII tubulin (TuJ1) to assess total intraepidermal nerve fiber (IENF) density of glabrous skin of the hindpaw footpads. Similar to Advillin-Tsc2 mice, Nav-Tsc2 mice showed a significant reduction of total IENF density (*p* < 0.0001; Fig. 3*A–C*). We also observed aberrant morphology of fibers similar to previously reported (Fig. 3*B*, arrowhead; Abe et al., 2010). As Cre expression occurs perinatally from the Nav1.8 transgene in contrast to embryonic day 12.5 expression of Advillin-Cre (Agarwal et al., 2004; Hasegawa et al., 2007), these data suggest that mTORC1 activation after embryonic development is sufficient to affect peripheral target innervation.

**Figure 4.**
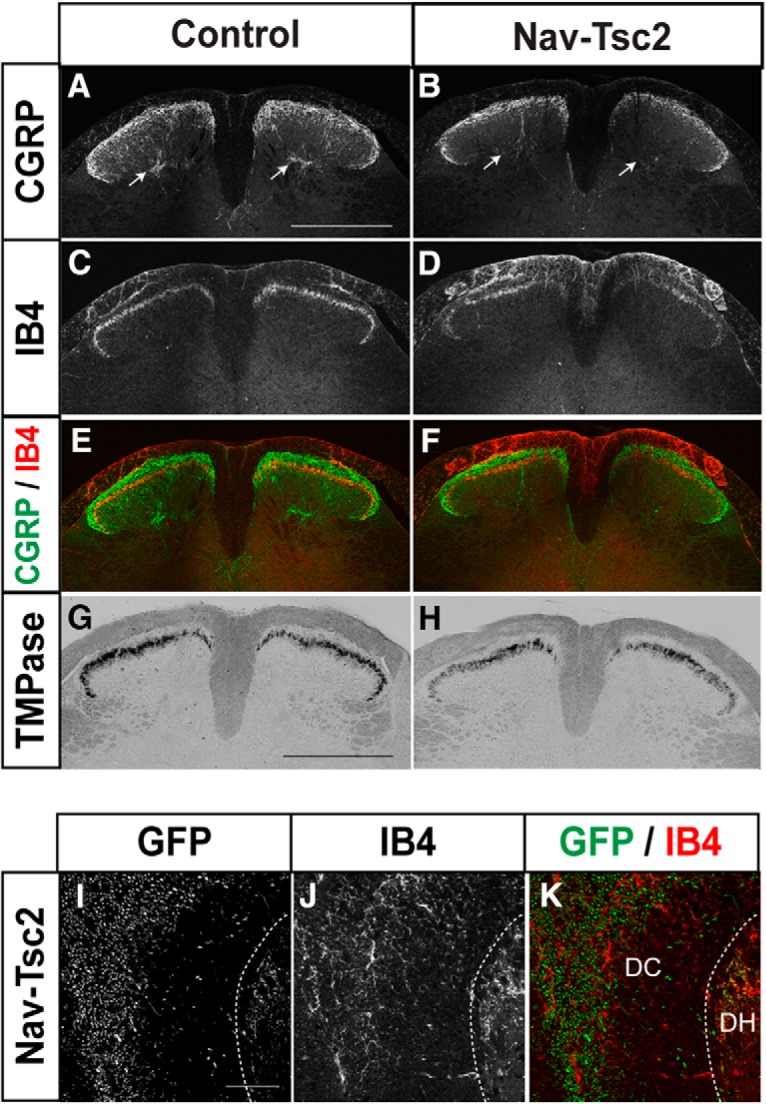
Laminar targeting of peptidergic and nonpeptidergic fibers is normal in dorsal horn of Nav-Tsc2 mice. ***A****–****F***, Control and Nav-Tsc2 lumbar spinal cord transverse sections labeled with CGRP (***A***, ***B***) or IB4 (***C***, ***D***) as well as merged images (***E***, ***F***). Note ectopic binding of IB4 in dorsal white matter of Nav-Tsc2 mice (***D***, ***F***). Arrows show deep lamina innervation by CGRP. Scale bar: 500 µm; *N* = 7. ***G***, ***H***, TMP histochemistry of control and Nav-Tsc2 lumbar spinal cord transverse sections. Scale bar: 500 µm; *N* = 11. ***I****–****K***, High-magnification confocal micrographs of lumbar spinal cord transverse sections from Nav-Tsc2; Rosa-GFP mice imaged for GFP fluorescence and IB4 labeling. Note lack of colocalization in the dorsal column (DC) white matter compared to dorsal horn (DH) gray matter. Scale bar: 50 µm; *N* = 5.

The central processes of C-nociceptors project to superficial lamina of the spinal cord dorsal horn. Specifically, peptidergic (CGRP-positive) C-nociceptors project to Lamina I and outer Lamina II (II_o_) while nonpeptidergic (IB4-positive) C-nociceptors project to inner Lamina II (II_i_; Fig. 4*A*,*C*,*G*). To determine whether DRG neuron fiber density was disrupted in the dorsal horn by Tsc2 deletion, we crossed a floxed *Rosa-GFP* reporter into control (specifically, *Nav1.8^Cre/+^; Tsc2^fl/+^*) and Nav-Tsc2 mice. Using IB4 as a marker of Lamina II, we analyzed the percentage area occupied by GFP signal in Laminas I and II. We observed a reduction in GFP-positive area in both dorsal horn lamina in Nav-Tsc2; Rosa-GFP mice compared to control; Rosa-GFP mice (Lamina I: *p* = 0.0403; Lamina II: *p* = 0.0002; Fig. 3*D–F*), suggesting target innervation is disrupted in both the peripheral and central branches of C-fiber neurons.

Despite the reduction in dorsal horn fiber density in Nav-Tsc2 mice, the proper targeting of each class of C-nociceptor to its appropriate superficial lamina of the lumbar spinal cord was unaffected (Fig. 4*A–F*). In addition to superficial lamina, CGRP-positive fibers project to deep laminae (Fig. 4*A*, arrows). Strong reduction of CGRP innervation to deep laminae was observed in all Nav-Tsc2 mice compared to control mice (Fig. 4*A*,*B*, arrows); however, reduced expression levels of CGRP may contribute to this phenotype.

In analyzing nonpeptidergic innervation of the lumbar spinal cord, we surprisingly observed ectopic IB4 binding that presented as filamentous or axon-like labeling in the dorsal white matter of the spinal cord in all Nav-Tsc2 mice, a phenotype never seen in control animals (Fig. 4*D*,*F*). IB4 is a lectin and can potentially bind to multiple proteins. Ectopic binding may result from upregulation of a binding target in that region or from mistargeting of nonpeptidergic axons into the white matter. TMPase is a marker for predominantly nonpeptidergic neurons that is known to colocalize extensively with IB4 in DRG and is present in DRG cell bodies as well as dorsal horn axon terminals (Bennett et al., 1998; Zylka et al., 2008). We performed TMP histochemistry on spinal cord sections to determine whether ectopic IB4 binding in the spinal cord of Nav-Tsc2 mice is a result of aberrant targeting of nonpeptidergic nociceptor axons. We did not observe TMPase-positive nonpeptidergic axon terminals outside of the dorsal horn superficial laminae in Nav-Tsc2 mice (Fig. 4*G*,*H*), suggesting that targeting of nonpeptidergic axon terminals was normal in these mice. To further investigate the nature of the ectopic IB4 labeling, we crossed a floxed *Rosa-GFP* reporter into control and Nav-Tsc2 mice. GFP signal was strong in axons of control; Rosa-GFP and Nav-Tsc2; Rosa-GFP mice in both gray and white matter (Figs. 3*D*,*E*, 4*I–K* ), consistent with GFP expression driven in ∼40% of myelinated DRG neurons some of which project to the white matter (Shields et al., 2012). However, IB4 labeling was interspersed between GFP signal in regions of the dorsal white matter, and colocalization of filamentous IB4 labeling with GFP fluorescence was not seen in lateral dorsal white matter (Fig. 4*I–K*). We conclude that ectopic IB4 labeling in spinal cord white matter of Nav-Tsc2 mice does not result from aberrant targeting of nonpeptidergic axons.

### Tsc2 is required for the full expression of peptidergic markers and ion channels in nociceptors

**Figure 5.**
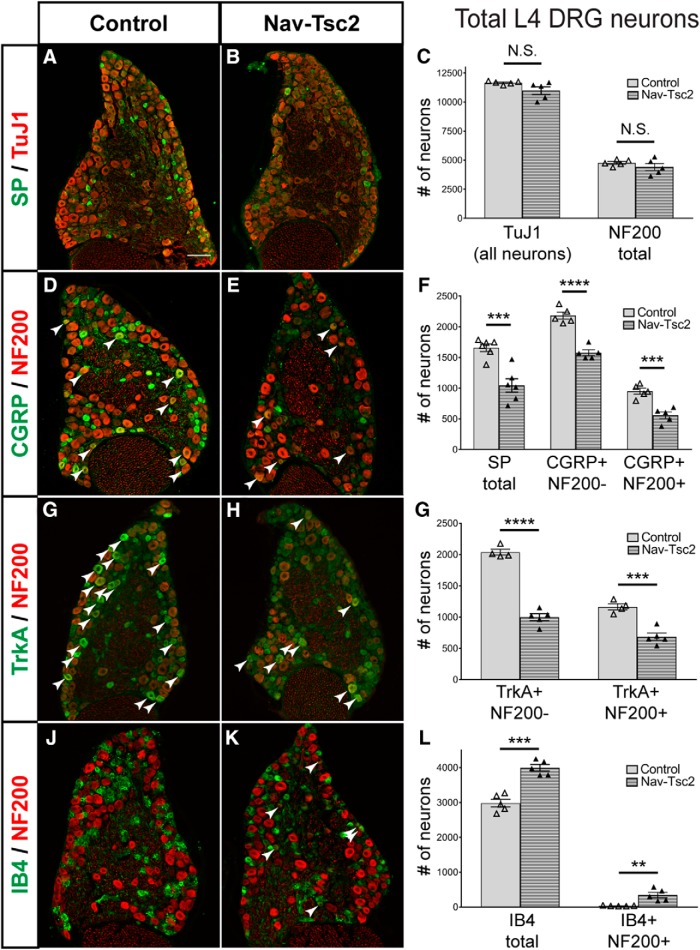
Peptidergic nociceptor markers are reduced in DRG from Nav-Tsc2 mice. Immunohistochemisty of adult L4 DRG transverse sections stained for SP and TuJ1 (***A***, ***B***) or NF200 and CGRP (***D***, ***E***), NF200 and TrkA (***G***, ***H***), or NF200 and IB4 (***J***, ***K***). Scale bar: 100 µm. Arrows point to neurons that colabeled with NF200. *C*, *F*, *G*, *L*, Total number of L4 DRG neurons labeling with markers as indicated. Individual animals plotted with mean ± SEM; *N* = 4-6; N.S. *p* > 0.05, **p < 0.01, ***p < 0.001, ****p < 0.0001.

Reduced nociceptor target innervation in Nav-Tsc2 mice could result from several possible mechanisms. One such mechanism is a reduction in the number of DRG neurons that project to those targets. To explore this possibility, we counted total neurons as well as presumptive myelinated neurons in adult L4 DRG of control and Nav-Tsc2 mice using antibodies directed against TuJ1 and NF200, respectively. Loss of Tsc2 resulted in no change in the total neuron number nor in the number of NF200-positive neurons (TuJ1: *p* = 0.081; NF200: *p* = 0.314; Fig. 5*A–E*).

To analyze the effects of Tsc2 deletion on specific nociceptor populations, we counted peptidergic nociceptors in L4 DRG that are immunoreactive for Substance P (SP), CGRP, and TrkA. Despite a differential regulation of mRNA expression, we observed similar phenotypes by immunohistochemistry for all three markers. For each, we observed a decrease in the intensity of immunoreactivity in DRG neurons from Nav-Tsc2 mice, however these neurons were still identifiable (Figs. 1*D*,*E*, 5*A*,*B*,*D*,*E*,*G*,*H* ). The number of SP neurons was reduced in Nav-Tsc2 DRG compared to controls (*p* = 0.0006; Fig. 5*A*,*B*,*F*). CGRP and TrkA neurons can project Aδ- or C-fiber axons, which are distinguishable by NF200 expression. Reductions were noted in the number of both NF200-negative and NF200-positive CGRP neurons as well as TrkA neurons in Nav-Tsc2 DRG compared to controls (CGRP+,NF200−: *p* < 0.0001; CGRP+, NF200+: *p* = 0.0007; TrkA+, NF200-: *p* < 0.0001; TrkA+,NF200+: *p* = 0.0005; Fig. 5*D–G*). While the number of peptidergic neurons may be underestimated due to decreased expression levels of these markers, these data suggest a strong reduction in phenotypic markers of peptidergic nociceptors.

10.1523/ENEURO.0436-17.2018.f7-1 Extended Data Figure 7-1 RNA-seq analysis of FACS-sorted neurons from control; Rosa-GFP and Nav-Tsc2; Rosa-GFP summarized in Figure 7. Red and blue text denotes upregulated and downregulated genes, respectively, in Nav-Tsc2 DRG compared to control (adjusted *p* < 0.05, log2 fold change >0.5 or <-0.5). Black text denotes no change in expression; padj denotes adjusted *p* value. Download Figure 7-1, DOCX file.

As peptidergic nociceptor number was reduced and the total number of neurons was unaffected in Nav-Tsc2 mice, we predicted an expansion of other populations of DRG neurons. Consistently, we noted a 34% increase in the average number of IB4 neurons in the DRG of Nav-Tsc2 mice compared to controls (*p* = 0.0001; Fig. 5*J–L*). Colabeling of IB4 and NF200 was rarely observed in control animals, however to our surprise, we identified a notable population of neurons that were positive for both IB4 and NF200 in Nav-Tsc2 mice (*p* = 0.0037; Fig. 5*J–L*, arrows). The size of this novel population accounted for the loss of NF200-positive CGRP or TrkA neurons and comprises 8.6% of the total IB4 population. As we did not observe ectopic IB4 or TMP labeling in deep laminae of the dorsal horn of Nav-Tsc2 mice (Fig. 4*D*,*H*), these supernumerary IB4-positive neurons may project to Lamina II_i_, the normal target of IB4-positive afferents. Together, these data show a gain of IB4-positive DRG neurons at the expense of peptidergic nociceptors in Nav-Tsc2 mice.

**Figure 6.**
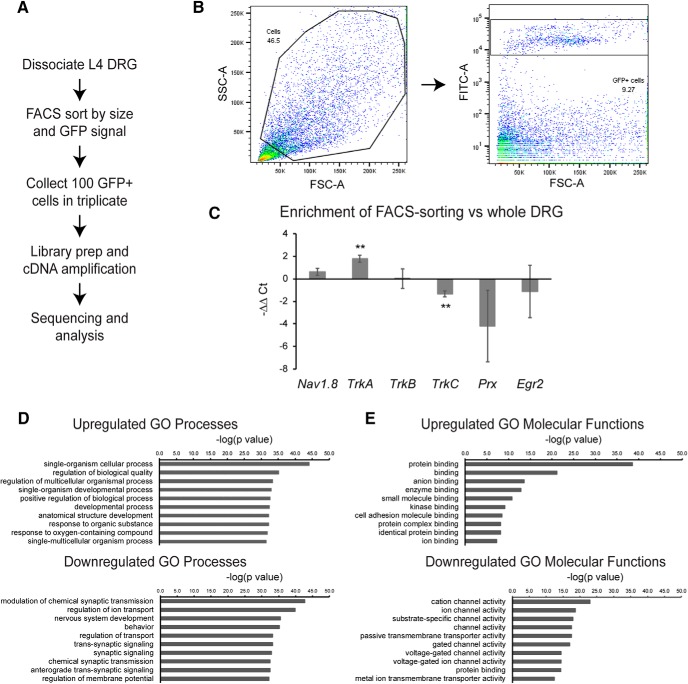
Transcriptional profiling of nociceptors reveals reduction of ion channel expression in Nav-Tsc2 DRG. ***A***, Experimental design for transcriptional profiling. ***B***, After gating by forward and side scatter, FITC-positive events were purified. ***C***, qPCR comparison of normalized neuronal and glial markers from FACS-sorted cells relative to whole DRG. ***D***, ***E***, Top 10 GO processes (***D***) and molecular functions (***E***) by statistical significance that were upregulated and downregulated in Nav-Tsc2 DRG as determined by *p* value; **p < 0.01.

To gain insight into the gene expression changes in DRG neurons resulting from Tsc2 deletion, we performed transcriptional profiling of neurons from control; Rosa-GFP and Nav-Tsc2; Rosa-GFP mice. L4 DRG neurons from these mice were dissociated, and GFP-positive cells were sorted by flow cytometry. Libraries were prepped from 100 GFP-positive cells and subjected to RNA-seq analysis (Fig. 6*A*,*B*). To determine the relative enrichment of nociceptors by FACS-sorting, we performed qPCR on control whole DRG and FACS-sorted GFP-positive cells from control DRG with markers of different neuron populations as well as glial markers. *TrkA* and *Nav1.8* expression was determined to denote nociceptors, while *TrkB* and *TrkC* were assayed for mechanoreceptors and proprioceptors, respectively (Usoskin et al., 2015). Periaxin (*Prx*) and *Egr2* (*Krox20*) were assayed as glial markers (Jones et al., 2007; Svaren and Meijer, 2008). We noted >3-fold increase in *TrkA* and a trend toward a 1.5-fold increase in normalized levels of *Nav1.8* in FACS-sorted samples relative to whole DRG (*TrkA*: *p* = 0.0014; *Nav1.8*: *p* = 0.0613; Fig. 6*C*). While we observed no change in the relative levels of *TrkB*, we observed a 60% reduction in normalized levels of *TrkC* in FACS-sorted samples compared to whole DRG (*TrkB*: *p* = 0.9758; *TrkC*: *p* = 0.002; Fig. 6*C*). Glial markers *Prx* and *Egr2* exhibited an average ∼16-fold and ∼2-fold reduction in FACS-sorted samples respectively, however these changes did not attain statistical significance due to high intersample variability (*Prx*: *p* = 0.178; *Egr2*: *p* = 0.5834; Fig. 6*C*). Together, this suggests that our FACS-sorted samples are highly enriched for nociceptors, with some glial and other neuron contamination.

**Figure 7.**
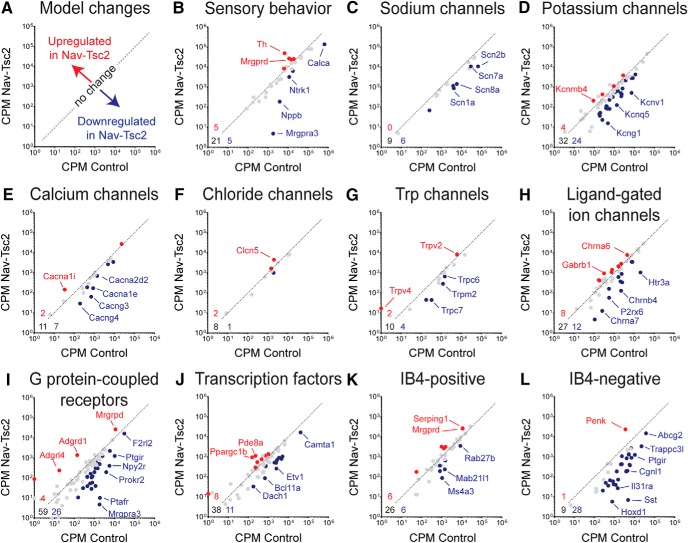
Tsc2 is required for full expression of ion channels and markers of IB4-negative DRG neurons. ***A***, Model of expression changes in Nav-Tsc2 mice. ***B****–****L***, Scatterplots of normalized counts in control and Nav-Tsc2 mice for candidate genes involved in sensory behavior (***B***), ion channels (***C–H***), G protein-coupled receptors (***I***), transcription factors (***J***), and the most enriched genes in IB4-positive (***K***) and IB4-negative (***L***) neurons expressing Nav1.8-Cre (Chiu et al., 2014). Red and blue points denote upregulated and downregulated genes, respectively, in Nav-Tsc2 DRG compared to control (adjusted *p* < 0.05, log2 fold change >0.5 or <-0.5). Gray points denote no change in expression. Gene lists and values can be found in Extended Data Figure 7-1. Select genes that were highly differentially expressed in each category are highlighted. Numbers in bottom left corner of panels denote number of upregulated (red), downregulated (blue), and unchanged (black) genes analyzed. Dotted line is representative plot of y = x for reference purposes only.

We then performed Gene Ontology (GO) analysis on the genes differentially expressed in Nav-Tsc2; Rosa-GFP compared to control; Rosa-GFP DRG (adjusted *p* < 0.05, log2 fold change >0.5 or <-0.5) and found that many of the downregulated categories of GO processes and molecular functions with the highest statistical significance relate to ion channel expression (Fig. 6*D*). To understand transcriptional shifts in ion channel expression in Nav-Tsc2 mice (Fig. 7*A*), we used the gene lists from a previously published study that also used FACS-sorting of DRG neurons expressing a fluorescent reporter in an Nav1.8-Cre-dependent manner (Chiu et al., 2014). This study identified genes in a number of different categories that are expressed in DRG neurons. We noted reduced expression of *Calca* (*CGRPa*) and *Ntrk1* (*TrkA*), consistent with our cell counting results, as well as several sensory behavior mediators of itch including *Nppb* and *Mrgpra3* (Fig. 7*B*). In DRG from Nav-Tsc2; Rosa-GFP mice, there was a preference toward downregulation of a number of sodium, potassium and calcium channels as well as G protein-coupled receptors (Fig. 7*C–E*,*I*). Other categories, including chloride, Trp and ligand-gated ion channels as well as transcription factors, showed a more random distribution of upregulated and downregulated genes in DRG from Nav-Tsc2; Rosa-GFP mice compared to control; Rosa-GFP (Fig. 7*F–H*,*J*). Differential expression data for individual genes can be found in the Extended Data Figure 7-1.

The previous report of DRG transcriptional profiling identified the 40 most enriched genes in IB4-positive and IB4-negative subgroups of Nav1.8-positive neurons (Chiu et al., 2014). As Nav-Tsc2 mice have an increase in the number IB4-positive neurons at the expense of IB4-negative ones, we analyzed the enriched gene sets from Chiu et al., to determine whether these genes are enriched in DRG from Nav-Tsc2 mice. Although we noted an increase in the number of IB4-positive neurons in DRG from Nav-Tsc2 mice (Fig. 5*J–L*), the expression of most genes previously shown to be enriched in IB4-positive neurons was unchanged in Nav-Tsc2 mice, and an equal number of genes were up- and downregulated (Fig. 7*K*). In contrast, we observed a reduction in the expression of the peptidergic markers SP, CGRP and TrkA by immunohistochemistry (Fig. 5), and also noted a strong reduction in the expression of genes enriched in the IB4-negative group. Of the 38 IB4-negative enriched genes found in our dataset, 28 were downregulated while only one was upregulated in DRG from Nav-Tsc2 mice (Fig. 7*L*). Together, this suggests notable deficits in peptidergic nociceptors in Nav-Tsc2 mice that may affect their function.

### Nav-Tsc2 mice have reduced sensitivity to noxious heat and decreased nerve injury-induced cold hypersensitivity

**Figure 8.**
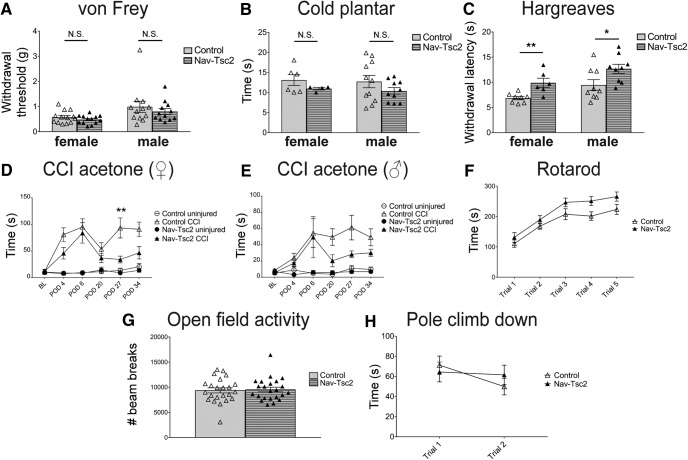
Nav-Tsc2 mice shows normal responses to mechanical and cold stimuli but have decreased heat sensitivity and injury-induced cold hypersensitivity. ***A***–***C***, Sensory behavior analysis of control and Nav-Tsc2 mice. ***A***, Withdrawal threshold for control and Nav-Tsc2 male and female mice using von Frey test; *N* = 12–13. ***B***, Latency to paw withdrawal from cold stimulus; *N* = 4–11. ***C***, Latency to paw withdrawal in Hargreaves test. Individual animals plotted with mean ± SEM; *N* = 9–13. ***D***, ***E***, Time spent in spontaneous pain behavior as a result of acetone application to paw of uninjured (circles) or injured (triangles) hindpaw at baseline and indicated time after CCI for female (***D***) and male (***E***) Nav-Tsc2 mice (closed shapes) and their control littermates (open shapes). Plotted as mean ± SEM; *N* = 8–11. BL, baseline. POD, post-operative day. ***F****–****H***, Sensorimotor battery showed no difference between control and Nav-Tsc2 mice. ***F***, Time to fall off an accelerating Rotarod was measured for Nav-Tsc2 mice (closed) and their control littermates (open). Data are graphed for each of five trials; *N* = 17. ***G***, Open field locomotor behavior was assessed over 1 h in Nav-Tsc2 mice and their control littermates with individual data points plotted; *N* = 23. ***H***, Time required to climb down a pole was measured for Nav-Tsc2 mice (closed) and their control littermates (open). Data graphed for each of two trials; *N* = 22. Data in ***G***, ***H*** shown as mean ± SEM; N.S. *p* > 0.05, *p < 0.05, **p < 0.01.

As nociceptors from Nav-Tsc2 mice showed altered target innervation and altered expression of some genes related to nociceptor phenotypes and sensory behaviors, we evaluated sensory behavior in these mice including sensitivity to heat, cold and mechanical stimuli. As a result of reduced expression of markers of peptidergic nociceptors, we hypothesized that Nav-Tsc2 mice might exhibit a phenotype similar to mice in which CGRP neurons are ablated (i.e., with reduced sensitivity to heat and cold hypersensitivity; McCoy et al., 2013). We analyzed male and female mice independently due to gender-dependent differences in sensory thresholds. Using the von Frey test for mechanical sensitivity, we observed no differences in the withdrawal threshold between control and Nav-Tsc2 mice (female: *p* = 0.2513; male *p* = 0.462; Fig. 8*A*). We also observed no differences in response to a cold stimulus between control and Nav-Tsc2 mice in the cold plantar assay or the acetone test (female: *p* = 0.239; male *p* = 0.2102; Fig. 8*B*,*D*,*E*). In contrast, both male and female Nav-Tsc2 mice displayed a decrease in sensitivity to noxious heat compared to control animals (female: *p* = 0.0046; male *p* = 0.0337; Fig. 8*C*), consistent with our prediction. To confirm that decreased sensitivity was not due to altered sensorimotor behavior, control and Nav-Tsc2 mice were subjected to a battery of tests to evaluate motor function. Control and Nav-Tsc2 mice were not distinguishable in any test performed, and therefore we report several representative tests. Nav-Tsc2 mice displayed a trend toward a better performance on the accelerating Rotarod compared to controls (*F* = 4.08, *p* = 0.0518; Fig. 8*F*). Additionally, no significant differences were noted between control and Nav-Tsc2 mice in open field activity (*p* = 0.8027; Fig. 8*G*) or in the pole test which evaluates performance of a complex motor task (*F* = 0.0425, *p* = 0.8376; Fig. 8*H*). Motor behavior of Nav-Tsc2 mice was indistinguishable from controls, indicating that changes in sensory behavioral responses were not due to motor impairment. mTORC1 inhibition by rapamycin injection has been shown to reduce mechanical hypersensitivity in chronic pain models such as spared nerve injury (Jiménez-Díaz et al., 2008; Geranton et al., 2009); however, similar analysis has not been performed in the context of constitutive mTORC1 activation specifically in nociceptors. Due to the previously reported requirement for mTORC1 activation and local protein translation for the full expression of nerve-injury-induced pain behaviors (Khoutorsky and Price, 2018), we expected to see increased nerve injury-induced hypersensitivity in Nav-Tsc2 mice. To determine whether mTORC1 activation affects nerve injury-induced hypersensitivity, we analyzed cold hypersensitivity induced by chronic constriction injury (CCI) of the sciatic nerve. We initially measured CCI-induced mechanical hypersensitivity and CCI-induced cold hypersensitivity in Nav-Tsc2 mice and control mice using the von Frey test and the acetone test, respectively. We observed inconsistent CCI-induced mechanical hypersensitivity in Nav-Tsc2 mice and control mice. However, both Nav-Tsc2 mice and control mice developed robust and stable cold hypersensitivity following CCI. We therefore used the acetone test as a measure of CCI-induced hypersensitivity. Cold sensitivity as measured by the acetone test was not affected at baseline in Nav-Tsc2 mice (Fig. 8*D*,*E*). While the acute phase of hypersensitivity in the first week following CCI was not affected by Tsc2 deletion, we observed a statistically significant reduction of nerve injury-induced cold hypersensitivity for females and a trend toward similar attenuation in male Nav-Tsc2 mice in the chronic phase several weeks after injury (female: *F* = 9.032, *p* = 0.0089; male: *F* = 3.403, *p* = 0.0807; Fig. 8*D*,*E*), contrary to our prediction. These data suggest that Tsc2 deletion and consequent chronic mTORC1 activation attenuates nerve injury-induced cold hypersensitivity.

## Discussion

In the present study, we analyzed the effects of Tsc2 deletion and chronic activation of mTORC1 in sensory neurons, primarily nociceptors, on peripheral and central target innervation, gene expression and sensory behavior. Consistent with the well-defined role of Tsc2 and mTORC1 in regulating cell size, we found an increase in cell soma size and axon diameter of C-fiber nociceptors in Nav-Tsc2 mice compared with nociceptors in control mice. To our surprise, we also found that Tsc2 deletion in nociceptors resulted in phenotypic changes in subpopulations of nociceptors, which manifested as a decrease in the number of peptidergic nociceptors with a concomitant increase in the number of IB4-positive neurons (presumably nonpeptidergic nociceptors). In addition, we found that deletion of Tsc2 and consequent chronic mTORC1 activation in sensory neurons resulted in decreased sensitivity to noxious heat as well as decreased nerve injury-induced cold hypersensitivity in Nav-Tsc2 mice. Together, these data show that Tsc2 functions to control target innervation and is required for full expression of nociceptor phenotypes and the expression of sensory-related genes. Disruption of Tsc2 in sensory neurons also results in abnormal sensory behavior, which may be due to the observed phenotypic changes in DRG neurons in which Tsc2 is deleted.

### Cell size and axon diameter are regulated by Tsc2 in C-nociceptors

mTORC1 is a well-characterized regulator of cell size (Fingar et al., 2002). Patients with Tuberous Sclerosis Complex (Tsc) complex have a chronic increase in mTORC1 signaling as a result of genetic disruption of Tsc1 or Tsc2. In mouse models, deletion of Tsc1 generated enlarged neuronal somata in hippocampal and cortical neurons (Tavazoie et al., 2005; Meikle et al., 2007). Consistent with these prior studies, our results show that cell size of unmyelinated peripheral sensory neurons is also increased in Tsc2-deleted mice, although we observed no effect on CGRP-positive presumptive myelinated neurons. It is possible that A-nociceptors normally have relatively high levels of an activated form of mTORC1 (Jiménez-Díaz et al., 2008; Geranton et al., 2009; Obara et al., 2015), which may result in increased baseline activity levels that make them less sensitive to constitutive mTORC1 signaling. Alternatively, A-nociceptors may engage compensatory mechanisms following Tsc2 deletion that maintain normal levels of cellular metabolism and/or cell morphology.

Peripheral axon diameter of C-fibers was increased in addition to soma size in Nav-Tsc2 mice. A similar increase in axon diameter was also observed in corpus callosum of mice with neuron-specific Tsc1 deletion (Ercan et al., 2017). Neuron-specific Tsc1 deletion also results in hypomyelination as a result of increased connective tissue growth factor (CTGF) expression (Meikle et al., 2007; Ercan et al., 2017). Similar to hypomyelination in a mouse model, white matter defects have also been noted in TSC patients (Ridler et al., 2001). We did not analyze myelination in the Nav-Tsc2 mice because Tsc2 was deleted primarily in unmyelinated C-nociceptors and only a subset of myelinated neurons. We did however observe disorganized bundles of C-fiber axons associated with Remak Schwann cells. We observed fewer axons per Remak bundle in adult but not in P29 Nav-Tsc2 animals when sorting axons by size is completed. It is possible that as Nav-Tsc2 axons continue to grow inside bundles after the completion of radial sorting, the Remak Schwann cell undergoes hypertrophy to compensate for increased axon volume or extrudes axons to maintain its size. Alternatively, Remak Schwann cells may undergo fragmentation or elaboration of processes, which was recently reported in injured nerves (Gomez-Sanchez et al., 2017). Remak Schwann cell hyperplasia may facilitate increased numbers of Remak Schwann cells to rebundle extruded axons, or if axons are larger than one micron in diameter they may become wrapped by myelinating Schwann cells. Future studies examining neuron-glia interactions in older mice with sensory neuron deletion of Tsc2 are required to determine whether this phenotype is progressive or stably maintained.

### Tsc2 deletion disrupts nociceptor target innervation, gene expression, and sensory behavior

Previous studies using pharmacological approaches have found that inhibition of mTORC1 signaling in the periphery, spinal cord or brain can attenuate bone cancer pain as well as inflammation- and nerve injury-induced pain (Price et al., 2007; Jiménez-Díaz et al., 2008; Geranton et al., 2009; Asante et al., 2010; Obara et al., 2011; Ferrari et al., 2013; Liang et al., 2013; Jiang et al., 2016; Kwon et al., 2017). These previous reports indicate that mTORC1 signaling at a peripheral and/or central locus within the pain transmission pathway is required for the full expression of these pain states. Conversely, 4EBP1 knockout mice, which would mimic global constitutive activation of mTORC1 on a single downstream effector, exhibit mechanical hypersensitivity (Khoutorsky et al., 2015). We thereby hypothesized that chronic mTORC1 activation would promote hypersensitivity to sensory stimuli. On the contrary, we found that chronic activation of mTORC1 in sensory neurons results in decreased sensitivity to noxious heat in naïve mice and decreased nerve injury-induced cold hypersensitivity, suggesting that chronic activation of mTORC1 in nociceptors may decrease pain.

There are a number of important differences between the current study and previous studies that have employed a pharmacological or global knockout approach to modulate mTORC1 signaling. First, we used a genetic strategy to specifically activate mTORC1 in sensory neurons, primarily nociceptors. The use of mTORC1 inhibitors in previous studies likely influences multiple cell types which may impact sensory behavior including primary sensory neurons, spinal neurons and cortical neurons as well as of a number of non-neuronal cells that may influence sensation including immune cells, Schwann cells, DRG satellite glia, and CNS glial cells (Geranton et al., 2009;; Asante et al., 2010; Ferrari et al., 2013; Nicks et al., 2014; Beirowski et al., 2017; Kwon et al., 2017). Inhibition of mTORC1 signaling in all or some of these diverse cell types simultaneously is likely to have a complex effect on sensory behavior. In addition to nociceptor specificity, our study chronically activated mTORC1 signaling. Most previous studies using mTORC1 inhibitors administered non-continuously and during a limited timeframe have found that mTORC1 inhibitors attenuate pain. There is evidence that chronic modulation of mTORC1 signaling may produce different effects compared with acute modulation. For example, chronic treatment of human patients or mice with mTORC1 inhibitors produces an increased incidence of pain (Budde et al., 2011; McCormack et al., 2011; Melemedjian et al., 2013). We report an attenuation in pain as a result of chronic mTORC1 activation in sensory neurons that express Nav1.8.

In Nav-Tsc2 mice, we observed disruptions in nociceptor soma and axon size, target innervation and gene expression including sensory behavior-related genes. Any or all of these changes may contribute to the attenuation of pain in Nav-Tsc2 mice. Cre expression in Nav1.8-Cre transgenic mice is initiated at embryonic day 17.5 (Agarwal et al., 2004), however cell fate determination of peptidergic and nonpeptidergic nociceptors is not complete until after the third postnatal week in mice (Molliver et al., 1997). Therefore, the sensory behavior changes observed in Nav-Tsc2 mice may be a result of developmental abnormalities in DRG neuron specification or a failure to maintain a nociceptor phenotype in maturity. Previous studies in which DRG phenotype specification was altered by perturbing expression of the transcription factor Runx1 in an Nav1.8-Cre-dependent manner resulted in changes in DRG target innervation and in sensory behavior (Chen et al., 2006; Abdel Samad et al., 2010; Yang et al., 2013). Attenuated pain responses in Nav-Tsc2 mice may similarly be a result of developmental anomalies. Innervation defects in Nav-Tsc2 mice may result from failure to innervate target tissue or from axon retraction. For instance, long-term maintenance of increased axon diameter in Nav-Tsc2 mice may pose a negative impact on axon health and morphology, which in turn may cause retraction from targets. The disruptions in axon morphology found in Nav-Tsc2 mice may underlie the observed sensory behavioral changes.

Nociceptor subpopulations have been correlated with modality-specific sensory responses (Cavanaugh et al., 2009; McCoy et al., 2013). CGRP-positive neurons are required for heat sensitivity, but also tonically suppress cold sensitivity (McCoy et al., 2013). Therefore, the observed hyposensitivity to noxious heat in Nav-Tsc2 mice is consistent with preferential disruption of CGRP neurons. Although we did not test responses to pruritogens in this study, we would also predict that responses to some pruritogens may be reduced due to decreased expression of *MrgprA3*, *Nppb*, and *Il31ra* (Liu et al., 2009; McCoy et al., 2013; Mishra and Hoon, 2013; Cevikbas et al., 2014). We did not observe a change in baseline cold sensitivity in these mice. Mechanical sensitivity, a modality that requires nonpeptidergic nociceptors (Cavanaugh et al., 2009), was unchanged in Nav-Tsc2 mice, consistent with the comparatively normal gene expression of these neurons. It is not yet clear whether changes in innervation density and gene expression are related to disrupted nociceptor development or maintenance, however they likely are contributors to reduced sensitivity in Nav-Tsc2 mice.

As mTORC1 is required for full expression of inflammatory and neuropathic pain states (Obara and Hunt, 2014; Lutz et al., 2015), we predicted that constitutive activation of the pathway would produce hypersensitivity in naïve mice. On the contrary, we observed normal mechanical and cold thresholds and decreased sensitivity to heat in naïve Nav-Tsc2 mice as well as decreased cold hypersensitivity after nerve injury. From these results, it is enticing to conclude that mTORC1 activation does not promote pain and may even reduce it. However, genetic mTORC1 activation induced complex changes in nociceptors from Nav-Tsc2 mice that may cooperate to generate the observed behaviors. It will be important to investigate the effects of chronic mTORC1 activation on sensory behavior in mice in which mTORC1 activation is initiated in adulthood using inducible Cre expression or pharmacological means. A recent study found that adult deletion of Pten, a negative regulator of mTORC1 activity farther upstream than Tsc2, did not alter baseline mechanical or thermal thresholds (Gallaher and Steward, 2018). However, the regeneration enhancement in these mice was very modest, suggesting that Pten may be a less potent regulator of the mTORC1 pathway in the peripheral nervous system than Tsc2. Adult deletion of Tsc2 in sensory neurons will be required to determine whether the Tsc2/mTORC1 signaling axis can be activated after nerve injury to enhance regenerative axon growth without stimulating pain.

## Synthesis

Reviewing Editor: Mike Fainzilber, Weizmann Institute of Science Decisions are customarily a result of the Reviewing Editor and the peer reviewers coming together and discussing their recommendations until a consensus is reached. When revisions are invited, a fact-based synthesis statement explaining their decision and outlining what is needed to prepare a revision will be listed below. The following reviewer(s) agreed to reveal their identity: Theodore Price. Note: If this manuscript was transferred from JNeurosci and a decision was made to accept the manuscript without peer review, a brief statement to this effect will instead be what is listed below.

This paper uses a genetic technique to delete Tsc2 early in development leading to increased mTORC1 activity specifically in nociceptive neurons in the DRG. The resulting phenotype is a change in the distribution and function of peptidergic nociceptors that has an impact on adult pain behaviors. The work is relevant to those in the development, pain neurobiology and nerve regeneration fields. While the manuscript is well written and the findings are potentially interesting, there are several issues that the authors should address:

1) It is a pity that the authors do not go in greater details regarding the mechanisms by which the activation of mTOR produces alterations in the nociceptive neurons populations. It would benefit the paper greatly if RNA-seq is done for DRGs of controls and Nav-Tsc2 mice to identify the general transcriptional shifts resulting from neuronal population changes in these animals.

2) The acetone test is not really a test for response to cold, it stimulates mild evaporative cooling which only really produces a strong response in mice with nerve injuries. A better test would the cold plate or the dry ice test developed by the Gereau group.

3) Can the authors do electrophysiology to check for changes in sensory conductance in the sciatic nerve in vivo? The differences in axons shown in E.M. would support that.

4) Specific experimental issues:

Figure 3 A-K. In the manuscript the authors state that “Tsc2 deletion produces a preferential loss of peptidergic intraepidermal nerve fibers” (page 17, 5th line from the bottom). This is implied because the average IENF loss is approximately equal to the average peptidergic loss. It would be advisable to verify this statement by direct quantification of non peptidergic innervation using the appropriate markers (e.g. IB4).

Figure 3E-L. CGRP innervation is determined on the basis of CGRP intensity over Tuj1. Interestingly, the authors state that “CGRP-positive neurons exhibited decrease intensity of immunostaining in nav-Tsc2 mice” (page 15, line 10 from the top). Could this affect the quantifications of figure 3E-L and 3A-K? The authors should consider this point carefully (maybe comparing the CGRP staining vs Tuj1 in DRG sections vs innervation), bearing in mind that CGRP staining in axons will be weaker than in the cell bodies.

Figure 4A-D. The authors should provide an appropriate quantification of the innervation as was done for the previous figure especially since they state that “TMP histochemistry confirmed a slight reduction of nonpeptidergic fiber innervation in the dorsal horn of Nav-Tsc2 mice compared to controls” (page 19, paragraph 2, line 4-5). Judging from figure 3L, the difference in innervation of IB4 neurons is non-significant and is hard to confirm a reduction by eye in figure 4A-B.

5) Issues requiring rewriting or changes in interpretation:

a) It is unlikely that mTOR is found in only one specific population of sensory neurons. The protein is ubiquitous, as shown by single cell RNA sequencing. Do the authors statements in the introduction actually mean that mTOR activation (measured by p-mTOR) seems to be higher in a population of A nociceptors and some C fibers? Please clarify.

b) There is a new review from Khoutorsky and Price in TiNS on translation regulation and pain that the authors may want to consider citing in the introduction (maybe to replace the Price and Geranton 2009 review).

c) Concluding that the Khoutorsky et al paper shows that this is entirely due to central sensitization is not very likely. It seems to be due to a gain of function of neuroligin expression which likely also requires some input from the DRG since those synapses seem to be prolifierating on spinal cord neurons.

d) The interpretation that chronic mTORC1 activation may decrease pain is very difficult to swallow. The existing evidence from neuropathic and inflammatory models is the opposite. Moreover, the model system here produces a profound change in the peptidergic nociceptor population that almost certainly has a strong effect on the behaviors that are being shown here. It is highly questionable that activation of mTORC1 in adult DRG neurons would lead to a similar phenotype.

e) The sentence “These normal mechanical and cold thresholds suggest that TSC2 deletion and mTORC1 activation alone is not sufficient to generate hypersensitivity” is unlikely to be true, for exactly the reasons that the authors state in the following sentences. The authors should reconsider this interpretation of their results, bearing in mind the developmental changes that are profoundly induced by mTORC1 activation throughout early development.

## Author Response

Detailed responses to reviewers.

Reviewers' comments are in black. Authors' responses are in red.

This paper uses a genetic technique to delete Tsc2 early in development leading to increased mTORC1 activity specifically in nociceptive neurons in the DRG. The resulting phenotype is a change in the distribution and function of peptidergic nociceptors that has an impact on adult pain behaviors. The work is relevant to those in the development, pain neurobiology and nerve regeneration fields. While the manuscript is well written and the findings are potentially interesting, there are several issues that the authors should address:

We thank the reviewers for their prompt and thoughtful reading of our manuscript and their constructive and detailed suggestions. We have performed extensive additional experiments that have increased the quality and depth of the paper.

1) It is a pity that the authors do not go in greater details regarding the mechanisms by which the activation of mTOR produces alterations in the nociceptive neurons populations. It would benefit the paper greatly if RNA-seq is done for DRGs of controls and Nav-Tsc2 mice to identify the general transcriptional shifts resulting from neuronal population changes in these animals.

We agree with the reviewer that an understanding of the general transcriptional shifts could help put other data in the manuscript into context. To achieve this, we enriched for Nav1.8-Cre-positive L4 DRG neurons using fluorescence-associated cell sorting (FACS) from control; Rosa-GFP and Nav-Tsc2; Rosa-GFP mice. Cells were collected and RNA-seq analysis was performed on 100 cells for each animal (3 technical replicates, N=4). As RNA-seq data is more sensitive than qPCR, we removed the qPCR data from the previous submission and added 2 figures to the manuscript that show the purification of neurons (Fig 6A-C), GO processes and molecular functions (Fig 6D-E), and changes in candidate gene expression in Nav-Tsc2 DRG (Fig 7, Extended Data). We used the list of candidate genes known to be expressed in DRG neurons described in Chiu et al as this study also FACS-sorted DRG neurons in an Nav1.8-Cre dependent manner (Chiu et al., 2014). Candidate genes with the most significant changes were highlighted in the figure, and we include Extended Data that reports log2 fold change, counts in control and Nav-Tsc2 mice, and adjusted p value for all genes presented in Fig 7. We have also uploaded FastQ and count files as well as DESeq2 analysis to an NCBI GEO database with the information noted in the manuscript (GSE112499).

A notable interpretation of the behavior phenotype was changed as a result of the RNA-seq analysis. Neither TrpA1 nor TrpV1 expression was affected in Nav-Tsc2 DRG by RNA-seq analysis. Our previous interpretations that reduced expression of these genes may contribute to the reduced heat sensitivity and injury-induced cold hypersensitivity have been removed from the manuscript.

2) The acetone test is not really a test for response to cold, it stimulates mild evaporative cooling which only really produces a strong response in mice with nerve injuries. A better test would the cold plate or the dry ice test developed by the Gereau group.

We agree and have performed a cold plantar assay with dry ice as per the Gereau lab publication using with N=10 male and N=4 female (Brenner et al., 2012). We noted no difference in response to cold stimulus in either male or female mice. We replaced the previous graph of acetone test with the data from the cold plantar test (Fig. 8B). Acetone response is still noted at the baseline time point in the chronic constriction injury study (Fig 8D-E).

3) Can the authors do electrophysiology to check for changes in sensory conductance in the sciatic nerve in vivo? The differences in axons shown in E.M. would support that.

We appreciate the reviewer's suggestion. While we agree that this would be an interesting experiment in light of the increased axon diameter and changes in ion channel expression in the Nav-Tsc2 mice, in vivo sensory conductance is not a technique that any of the authors' labs are able to perform. We feel, and we hope the reviewers agree, that our responses to the other points, especially inclusion of transcriptional profiling of FACS-purified nociceptors, are sufficient to merit publication of the manuscript.

4) Specific experimental issues:

We find the 3 individual points in this section all speak to a common theme. We address the points individually, and then conclude with our response to the issue of innervation as a whole.

Figure 3 A-K. In the manuscript the authors state that “Tsc2 deletion produces a preferential loss of peptidergic intraepidermal nerve fibers” (page 17, 5th line from the bottom). This is implied because the average IENF loss is approximately equal to the average peptidergic loss. It would be advisable to verify this statement by direct quantification of non peptidergic innervation using the appropriate markers (e.g. IB4).

We agree with the reviewer that having a positive marker of nonpeptidergic fibers would be useful. Unfortunately, IB4 staining is insufficient as staining of fibers in the skin is obscured by IB4 staining of keratinocytes (Rice, 1993; Zylka et al., 2005). TMP histochemistry also does not label IENFs. The field generally utilizes GFP reporter mice such as MrgprD-GFP or Ret-GFP to count nonpeptidergic IENFs, but we do not have access to these lines at this time. We removed our interpretation of nonpeptidergic fibers from the text. See below for our complete response.

Figure 3E-L. CGRP innervation is determined on the basis of CGRP intensity over Tuj1. Interestingly, the authors state that “CGRP-positive neurons exhibited decrease intensity of immunostaining in nav-Tsc2 mice” (page 15, line 10 from the top). Could this affect the quantifications of figure 3E-L and 3A-K? The authors should consider this point carefully (maybe comparing the CGRP staining vs Tuj1 in DRG sections vs innervation), bearing in mind that CGRP staining in axons will be weaker than in the cell bodies.

We thank the reviewer for this comment, and we agree that decreased intensity of CGRP immunoreactivity in DRG neurons confounds the interpretation of the innervation data in both the spinal cord and the skin. While we understand the point of the reviewer's suggested experiment, any result would at best indirectly address our intended purpose of assessing innervation in the Nav-Tsc2 mice. See below for response.

Figure 4A-D. The authors should provide an appropriate quantification of the innervation as was done for the previous figure especially since they state that “TMP histochemistry confirmed a slight reduction of nonpeptidergic fiber innervation in the dorsal horn of Nav-Tsc2 mice compared to controls” (page 19, paragraph 2, line 4-5). Judging from figure 3L, the difference in innervation of IB4 neurons is non-significant and is hard to confirm a reduction by eye in figure 4A-B.

Small changes in development time from slide to slide may induce variability of intensity in quantification of the TMP colorimetric assay. For this reason and others cited below, we chose to remove assessment of intensity in our characterization of TMP histochemistry. Our intent is to use this staining to confirm that nonpeptidergic fibers in the spinal cord do not terminate ectopically, and we have changed the corresponding text to reflect this point.

Response to all 3 comments: Each of the above points ask for experimental solidification of the data characterizing target innervation in the Nav-Tsc2 mice. A previous study utilized Advillin-Cre deletion of Tsc2, whereby Tsc2 is deleted at embryonic day 12.5 (Hasegawa et al., 2007), and those mice exhibited a defect in IENF density (Abe et al., 2010). We show that in Nav-Tsc2 mice where Tsc2 is deleted perinatally, there was a similar reduction of IENF density in glabrous skin. As raised by the reviewer, the reduction in CGRP immunoreactivity in Nav-Tsc2 DRG neurons may lead to a misinterpreted reduction in fiber number. We therefore removed the CGRP fiber density data from the manuscript. Our total IENF density data both replicates the previous work with a different Cre line as well as showing that Tsc2 deletion during embryogenesis is not required to observe the fiber density phenotype. Reporter lines could help to determine fiber density of nociceptor subtypes, but we are not equipped for such experiments at this time.

For spinal cord innervation, we analzyed fiber density instead of marker intensity. For the same reasons as stated above for IENFs, we removed intensity quantification for CGRP and IB4. To determine dorsal horn innervation density, we used the Rosa-GFP reporter to label all Nav1.8-Cre-positive fibers and stained sections for IB4 to mark lamina II. The dorsal horn region dorsal to IB4 labeling was denoted as lamina I. Confocal images were analyzed for the % area occupied by GFP signal to assess fiber density. We normalized this to the percentage of GFP-positive neurons in the L4 DRG for the same animals and set the average control value to 1. This data is now presented in Figure 3, where we conclude that there is a reduction in fiber density in both lamina I and lamina II of Nav-Tsc2 mice. We hope the reviewers agree that this data does a better job of showing a reduction in central innervation in the Nav-Tsc2 mice.

While the CGRP, IB4, and TMPase intensity may be confounding for quantitative purposes in the spinal cord, these images still provide qualitative value to the paper by demonstrating proper laminar targeting of both peptidergic and nonpeptidergic neurons, and our interpretation in the text has been altered to reflect this point. We have also improved the image showing ectopic IB4 binding in the white matter using higher magnification (Fig. 4I-K).

Last, to further support the loss of peptidergic nociceptor markers, we quantified the number of Substance P-positive neurons in the L4 DRG of control and Nav-Tsc2 mice. Whereas of CGRP and TrkA expression were both strongly reduced as determined by our RNA-seq analysis (Fig. 7) and visually by IHC (Fig. 5), Substance P expression was not affected by Tsc2 deletion according to RNA-seq (Fig 7, Extended data) or our qPCR (previous submission). We therefore analyzed Substance P expression in DRG by IHC. The changes in Substance P staining in DRG are largely similar to both CGRP and TrkA in both intensity and cell number. Substance P staining and quantification have been added to Figure 5.

5) Issues requiring rewriting or changes in interpretation:

a) It is unlikely that mTOR is found in only one specific population of sensory neurons. The protein is ubiquitous, as shown by single cell RNA sequencing. Do the authors statements in the introduction actually mean that mTOR activation (measured by p-mTOR) seems to be higher in a population of A nociceptors and some C fibers? Please clarify.

We agree with the reviewer's assessment that mTOR is ubiquitous, however the cited studies by Stephen Hunt's group have shown that mTOR phosphorylation seems to be more prevalent in A-fiber axons (Geranton et al., 2009; Jimenez-Diaz et al., 2008; Obara et al., 2011). They also contend that mTORC1 inhibition by rapamycin predominantly affects A-nociceptor modalities, and this is at least in part a result of axonal mTORC1 inhibition. We agree with the reviewer that mTORC1 is expressed in many cell types. As a result, it is possible that mTORC1 activity has both cell type- as well as subcellular localization-dependent roles in sensory behavior. We clarified these points in the text.

b) There is a new review from Khoutorsky and Price in TiNS on translation regulation and pain that the authors may want to consider citing in the introduction (maybe to replace the Price and Geranton 2009 review).

Thank you for making us aware of this review. We have updated the citation as suggested.

c) Concluding that the Khoutorsky et al paper shows that this is entirely due to central sensitization is not very likely. It seems to be due to a gain of function of neuroligin expression which likely also requires some input from the DRG since those synapses seem to be prolifierating on spinal cord neurons.

The reviewer is absolutely correct that our wording was an over-simplification of that study. Our intent was to highlight that the analysis in that paper was performed largely at the level of the spinal cord and in a global knockout, which is in contrast with the peripheral nature and targeted deletion of our study. We changed the description of that citation to better highlight our intent.

d) The interpretation that chronic mTORC1 activation may decrease pain is very difficult to swallow. The existing evidence from neuropathic and inflammatory models is the opposite. Moreover, the model system here produces a profound change in the peptidergic nociceptor population that almost certainly has a strong effect on the behaviors that are being shown here. It is highly questionable that activation of mTORC1 in adult DRG neurons would lead to a similar phenotype.

We also expected to see baseline hypersensitivity in the Nav-Tsc2 mice and agree that the changes in peptidergic nociceptors are likely to cause the behavioral phenotypes observed. We do see a strong reduction in the number of DRG neurons that stain with peptidergic markers in Nav-Tsc2 mice as well as a dramatic trend towards reduction of expression of genes enriched in the IB4-negative group described by Chiu et al 2014. Interestingly, and somewhat surprisingly, we did not observe cold hypersensitivity in Nav-Tsc2 mice as would be predicted from the CGRP cell ablation study (McCoy et al., 2013). Regardless, we favor the interpretation suggested by the reviewer. We revised the discussion to include the interpretation that the sensory behavior changes observed in Nav-Tsc2 mice may be a result of developmental abnormalities in DRG neuron specification or a failure to maintain a nociceptor phenotype in maturity. As an example, we reference studies using Nav1.8-Cre to modulate Runx1 transcription factor activity, which also identify nociceptor phenotype and innervation deficits. Future studies will be required to test whether Tsc2 deletion in adult neurons would lead to a similar phenotype as the Nav1.8-Cre mediated Tsc2 deletion. However, a recent study showed that adult deletion of PTEN did not alter baseline mechanical or thermal thresholds two weeks after AAV-Cre viral injection into floxxed mice (Gallaher & Steward, 2018). We have cited these studies in our discussion and reworded some of our interpretations to reflect the developmental changes.

e) The sentence “These normal mechanical and cold thresholds suggest that TSC2 deletion and mTORC1 activation alone is not sufficient to generate hypersensitivity” is unlikely to be true, for exactly the reasons that the authors state in the following sentences. The authors should reconsider this interpretation of their results, bearing in mind the developmental changes that are profoundly induced by mTORC1 activation throughout early development.

We agree with the reviewer, and we have reworded our interpretation. See also the response to the previous comment.

References cited in this response to reviewers:

Abe, N., Borson, S. H., Gambello, M. J., Wang, F., & Cavalli, V. (2010). Mammalian target of rapamycin (mTOR) activation increases axonal growth capacity of injured peripheral nerves. J Biol Chem, 285(36), 28034-28043. doi:10.1074/jbc.M110.125336

Brenner, D. S., Golden, J. P., & Gereau, R. W. t. (2012). A novel behavioral assay for measuring cold sensation in mice. PLoS One, 7(6), e39765. doi:10.1371/journal.pone.0039765

Chiu, I. M., Barrett, L. B., Williams, E. K., Strochlic, D. E., Lee, S., Weyer, A. D., . . . Woolf, C. J. (2014). Transcriptional profiling at whole population and single cell levels reveals somatosensory neuron molecular diversity. Elife, 3. doi:10.7554/eLife.04660

Gallaher, Z. R., & Steward, O. (2018). Modest enhancement of sensory axon regeneration in the sciatic nerve with conditional co-deletion of PTEN and SOCS3 in the dorsal root ganglia of adult mice. Exp Neurol, 303, 120-133. doi:10.1016/j.expneurol.2018.02.012

Geranton, S. M., Jimenez-Diaz, L., Torsney, C., Tochiki, K. K., Stuart, S. A., Leith, J. L., . . . Hunt, S. P. (2009). A rapamycin-sensitive signaling pathway is essential for the full expression of persistent pain states. Journal of Neuroscience, 29(47), 15017-15027. doi:10.1523/JNEUROSCI.3451-09.2009

Hasegawa, H., Abbott, S., Han, B. X., Qi, Y., & Wang, F. (2007). Analyzing somatosensory axon projections with the sensory neuron-specific Advillin gene. Journal of Neuroscience, 27(52), 14404-14414. doi:10.1523/JNEUROSCI.4908-07.2007

Jimenez-Diaz, L., Geranton, S. M., Passmore, G. M., Leith, J. L., Fisher, A. S., Berliocchi, L., . . . Hunt, S. P. (2008). Local translation in primary afferent fibers regulates nociception. PLoS One, 3(4), e1961. doi:10.1371/journal.pone.0001961

McCoy, E. S., Taylor-Blake, B., Street, S. E., Pribisko, A. L., Zheng, J., & Zylka, M. J. (2013). Peptidergic CGRPalpha primary sensory neurons encode heat and itch and tonically suppress sensitivity to cold. Neuron, 78(1), 138-151. doi:10.1016/j.neuron.2013.01.030

Obara, I., Tochiki, K. K., Geranton, S. M., Carr, F. B., Lumb, B. M., Liu, Q., & Hunt, S. P. (2011). Systemic inhibition of the mammalian target of rapamycin (mTOR) pathway reduces neuropathic pain in mice. Pain, 152(11), 2582-2595. doi:10.1016/j.pain.2011.07.025

Rice, F. L. (1993). Structure, vascularization, and innervation of the mystacial pad of the rat as revealed by the lectin Griffonia simplicifolia. J Comp Neurol, 337(3), 386-399. doi:10.1002/cne.903370304

Zylka, M. J., Rice, F. L., & Anderson, D. J. (2005). Topographically distinct epidermal nociceptive circuits revealed by axonal tracers targeted to Mrgprd. Neuron, 45(1), 17-25. doi:10.1016/j.neuron.2004.12.015
