# Supplementary material for: Deletion of Tsc2 in Nociceptors Reduces Target Innervation, Ion Channel Expression, and Sensitivity to Heat
Source: eNeuro. 2018 May 3;5(2):ENEURO.0436-17.2018. doi: 10.1523/ENEURO.0436-17.2018 (PMC5952427; doi:10.1523/ENEURO.0436-17.2018)
Supplement: Extended Data Figure 7-1 — RNA-seq analysis of FACS-sorted neurons from control; Rosa-GFP and Nav-Tsc2; Rosa-GFP summarized in Figure 7. Red and blue text denotes upregulated and downregulated genes, respectively, in Nav-Tsc2 DRG compared to control (adjusted p < 0.05, log2 fold change >0.5 or <-0.5). Black text denotes no change in expression; padj denotes adjusted p value. Download Figure 7-1, DOCX file. [file sup_enu-eN-NWR-0436-17-s01.docx]

**Extended Data Figure 7-1.**

| **Sensory behavior** |  |  |  |  |  |  |  |  |  |  |
| --- | --- | --- | --- | --- | --- | --- | --- | --- | --- | --- |
| MGI symbol | log2 fold change ± SEM | | | Control average counts ± SD | | | Nav-Tsc2 average counts ± SD | | | padj |
| Mrgpra3 | -7.033 | ± | 1.255 | 1782.45 | ± | 2134.45 | 4.71 | ± | 5.58 | 3.37E-07 |
| Nppb | -3.453 | ± | 0.871 | 3762.87 | ± | 2592.72 | 185.74 | ± | 147.83 | 0.00056 |
| Calca | -2.088 | ± | 0.244 | 645698.39 | ± | 240298.46 | 136680.66 | ± | 68535.80 | 7.30E-16 |
| Ntrk1 | -1.642 | ± | 0.218 | 11278.86 | ± | 2725.54 | 3183.35 | ± | 1541.08 | 2.08E-12 |
| Piezo2 | -1.180 | ± | 0.197 | 17207.54 | ± | 6457.53 | 6304.14 | ± | 2289.42 | 3.72E-08 |
| Ret | 0.746 | ± | 0.258 | 19175.41 | ± | 13296.67 | 25263.54 | ± | 8139.57 | 0.01694 |
| Trpv2 | 0.748 | ± | 0.125 | 6146.63 | ± | 1454.21 | 8433.98 | ± | 2870.24 | 3.87E-08 |
| Gfra3 | 1.101 | ± | 0.288 | 14421.46 | ± | 6073.49 | 23897.43 | ± | 8230.79 | 0.00093 |
| Mrgprd | 1.613 | ± | 0.573 | 11070.52 | ± | 10670.22 | 26817.07 | ± | 6593.87 | 0.02076 |
| Th | 3.089 | ± | 0.726 | 6621.69 | ± | 4451.31 | 49023.64 | ± | 32443.28 | 0.00018 |
| Mrgprx1 | -1.053 | ± | 0.782 | 354.02 | ± | 118.35 | 192.42 | ± | 275.65 | 0.34571 |
| Trpm8 | -0.883 | ± | 0.731 | 737.79 | ± | 697.41 | 310.71 | ± | 247.96 | 0.41016 |
| Trpv1 | -0.550 | ± | 0.244 | 14867.04 | ± | 5074.95 | 7949.72 | ± | 3611.81 | 0.07639 |
| Trpa1 | -0.499 | ± | 0.228 | 4399.03 | ± | 1456.45 | 2514.27 | ± | 962.42 | 0.08748 |
| Calcb | -0.487 | ± | 0.143 | 144379.27 | ± | 24433.68 | 80354.08 | ± | 28470.04 | 0.00385 |
| Ntrk2 | -0.397 | ± | 0.174 | 2663.93 | ± | 1193.62 | 1546.15 | ± | 473.95 | 0.07175 |
| Trpm3 | -0.292 | ± | 0.263 | 795.06 | ± | 356.03 | 533.54 | ± | 220.09 | 0.45893 |
| Scn9a | -0.282 | ± | 0.087 | 41670.40 | ± | 881.91 | 27927.59 | ± | 8495.78 | 0.00637 |
| Ntrk3 | -0.234 | ± | 0.266 | 3392.40 | ± | 918.41 | 2286.19 | ± | 837.15 | 0.57671 |
| Scn10a | -0.213 | ± | 0.120 | 44295.30 | ± | 12572.95 | 31340.67 | ± | 11257.89 | 0.18541 |
| Tac1 | -0.211 | ± | 0.416 | 109189.65 | ± | 70882.61 | 77695.91 | ± | 39219.47 | 0.77426 |
| Scn11a | -0.070 | ± | 0.226 | 22240.58 | ± | 12138.59 | 16825.47 | ± | 4773.52 | 0.87138 |
| Trpc1 | -0.003 | ± | 0.157 | 1421.45 | ± | 343.80 | 1095.38 | ± | 384.27 | 0.99261 |
| Slc17a8 | 0.252 | ± | 0.637 | 171.74 | ± | 96.30 | 172.02 | ± | 81.95 | 0.83083 |
| Tlx3 | 0.380 | ± | 0.307 | 84.41 | ± | 20.94 | 84.79 | ± | 39.04 | 0.39653 |
| Runx1 | 0.396 | ± | 0.200 | 6491.69 | ± | 2980.06 | 6536.78 | ± | 1428.67 | 0.13005 |
| Gfra1 | 0.405 | ± | 0.224 | 1314.21 | ± | 457.36 | 1356.48 | ± | 546.79 | 0.17485 |
| P2rx3 | 0.468 | ± | 0.195 | 14233.33 | ± | 6275.58 | 15333.29 | ± | 3837.39 | 0.0559 |
| Gfra2 | 0.604 | ± | 0.383 | 8241.50 | ± | 6383.38 | 10216.44 | ± | 3731.49 | 0.2514 |
| Hrh1 | 1.282 | ± | 0.710 | 184.99 | ± | 94.75 | 305.22 | ± | 73.10 | 0.17589 |

| **Sodium channels** |  |  |  |  |  |  |  |  |  |  |
| --- | --- | --- | --- | --- | --- | --- | --- | --- | --- | --- |
| MGI symbol | log2 fold change ± SEM | | | Control average counts ± SD | | | Nav-Tsc2 average counts ± SD | | | padj |
| Scn4a | -1.698 | ± | 0.626 | 253.06 | ± | 153.29 | 68.03 | ± | 25.40 | 0.02663 |
| Scn1a | -1.981 | ± | 0.402 | 4147.38 | ± | 1846.78 | 859.27 | ± | 436.15 | 9.95E-06 |
| Scn8a | -1.769 | ± | 0.295 | 5855.48 | ± | 740.80 | 1415.97 | ± | 874.06 | 3.83E-08 |
| Scn3b | -1.449 | ± | 0.196 | 3850.55 | ± | 431.45 | 1145.94 | ± | 519.08 | 5.57E-12 |
| Scn2b | -2.448 | ± | 0.299 | 67314.03 | ± | 26726.15 | 10778.63 | ± | 6398.10 | 1.38E-14 |
| Scn7a | -1.159 | ± | 0.119 | 29414.91 | ± | 3718.73 | 10718.73 | ± | 3602.14 | 2.49E-20 |
| Scn3a | -0.002 | ± | 0.264 | 811.64 | ± | 203.29 | 720.11 | ± | 341.21 | 0.99656 |
| Scn2a | 0.003 | ± | 0.175 | 1916.28 | ± | 600.52 | 1399.62 | ± | 325.25 | 0.99326 |
| Scn11a | -0.070 | ± | 0.226 | 22240.58 | ± | 12138.59 | 16825.47 | ± | 4773.52 | 0.87138 |
| Scn5a | -0.240 | ± | 0.407 | 561.03 | ± | 299.93 | 358.55 | ± | 198.16 | 0.7338 |
| Scn1b | -0.373 | ± | 0.238 | 624.88 | ± | 256.15 | 408.35 | ± | 160.66 | 0.25556 |
| Scnn1a | 3.748 | ± | 2.370 | 6.87 | ± | 12.89 | 4.91 | ± | 6.17 | 0.25006 |
| Scn10a | -0.213 | ± | 0.120 | 44295.30 | ± | 12572.95 | 31340.67 | ± | 11257.89 | 0.18541 |
| Scn4b | -1.306 | ± | 0.609 | 23996.02 | ± | 26497.03 | 8199.38 | ± | 2166.33 | 0.09519 |
| Scn9a | -0.282 | ± | 0.087 | 41670.40 | ± | 881.91 | 27927.59 | ± | 8495.78 | 0.00637 |

| **Potassium channels** |  |  |  |  |  |  |  |  |  |  |
| --- | --- | --- | --- | --- | --- | --- | --- | --- | --- | --- |
| MGI symbol | log2 fold change ± SEM | | | Control average counts ± SD | | | Nav-Tsc2 average counts ± SD | | | padj |
| Kcng1 | -4.823 | ± | 0.835 | 472.15 | ± | 337.45 | 15.40 | ± | 11.59 | 1.34E-07 |
| Kcnv1 | -3.883 | ± | 0.587 | 7131.71 | ± | 3460.53 | 508.58 | ± | 298.34 | 9.82E-10 |
| Kcnj2 | -3.213 | ± | 0.848 | 249.91 | ± | 293.14 | 22.75 | ± | 15.12 | 0.00105 |
| Kcnq5 | -3.194 | ± | 0.332 | 1322.57 | ± | 603.92 | 124.81 | ± | 83.90 | 6.84E-20 |
| Kcnc1 | -2.509 | ± | 0.450 | 569.36 | ± | 251.96 | 81.34 | ± | 37.51 | 3.82E-07 |
| Kcna1 | -2.429 | ± | 0.353 | 2199.80 | ± | 313.41 | 344.73 | ± | 195.38 | 1.66E-10 |
| Kcnq4 | -2.187 | ± | 0.571 | 435.21 | ± | 135.24 | 83.20 | ± | 18.71 | 0.00091 |
| Kcnt2 | -2.180 | ± | 0.252 | 2287.87 | ± | 444.11 | 432.68 | ± | 180.79 | 3.41E-16 |
| Kcnc3 | -2.118 | ± | 0.391 | 185.77 | ± | 60.39 | 36.80 | ± | 16.05 | 8.92E-07 |
| Kcnh2 | -1.769 | ± | 0.546 | 176.34 | ± | 89.89 | 44.68 | ± | 19.21 | 0.00631 |
| Kcna4 | -1.763 | ± | 0.223 | 2100.43 | ± | 560.06 | 494.80 | ± | 179.14 | 1.40E-13 |
| Kcnk4 | -1.716 | ± | 0.366 | 415.13 | ± | 256.41 | 109.10 | ± | 46.25 | 2.94E-05 |
| Kcnj16 | -1.710 | ± | 0.644 | 196.11 | ± | 172.95 | 51.64 | ± | 35.32 | 0.03083 |
| Kcns1 | -1.601 | ± | 0.562 | 381.59 | ± | 293.96 | 88.38 | ± | 25.26 | 0.01894 |
| Kcnk10 | -1.425 | ± | 0.504 | 395.24 | ± | 56.64 | 111.16 | ± | 54.52 | 0.01994 |
| Kcns3 | -1.232 | ± | 0.198 | 2528.49 | ± | 896.89 | 842.37 | ± | 306.35 | 1.08E-08 |
| Kcnab1 | -1.173 | ± | 0.464 | 8179.66 | ± | 6311.18 | 2780.97 | ± | 932.46 | 0.04166 |
| Kcnb1 | -1.119 | ± | 0.149 | 11094.04 | ± | 2303.50 | 4219.22 | ± | 1342.15 | 2.66E-12 |
| Kcnk2 | -1.070 | ± | 0.407 | 822.12 | ± | 212.09 | 316.61 | ± | 130.29 | 0.03239 |
| Kcnn2 | -1.032 | ± | 0.276 | 565.74 | ± | 152.87 | 229.46 | ± | 97.12 | 0.00123 |
| Kcna2 | -1.015 | ± | 0.163 | 7053.91 | ± | 1638.76 | 2802.16 | ± | 929.61 | 9.56E-09 |
| Kcnmb2 | -0.951 | ± | 0.334 | 1487.72 | ± | 1146.68 | 642.20 | ± | 293.22 | 0.01905 |
| Kctd10 | -0.665 | ± | 0.152 | 1081.47 | ± | 140.70 | 550.73 | ± | 174.35 | 0.00011 |
| Kcnd3 | -0.532 | ± | 0.209 | 3568.48 | ± | 781.57 | 2128.52 | ± | 959.90 | 0.03933 |
| Kcnh6 | 0.622 | ± | 0.203 | 925.31 | ± | 353.06 | 1116.82 | ± | 477.44 | 0.01052 |
| Kcnma1 | 0.893 | ± | 0.211 | 2700.81 | ± | 1248.31 | 3832.02 | ± | 945.61 | 0.00021 |
| Kctd15 | 1.231 | ± | 0.375 | 251.10 | ± | 191.84 | 426.99 | ± | 124.63 | 0.00553 |
| Kcnmb4 | 1.485 | ± | 0.306 | 89.12 | ± | 27.78 | 207.94 | ± | 80.94 | 1.43E-05 |
| Kcnd2 | -2.840 | ± | 1.354 | 59.35 | ± | 52.02 | 6.19 | ± | 7.59 | 0.1046 |
| Kcnh5 | -2.832 | ± | 1.215 | 73.40 | ± | 46.40 | 9.98 | ± | 10.74 | 0.06482 |
| Kcnk1 | -0.601 | ± | 0.297 | 665.14 | ± | 383.83 | 317.71 | ± | 164.02 | 0.12024 |
| Kcna6 | -0.397 | ± | 0.103 | 5273.82 | ± | 1061.82 | 3243.24 | ± | 1262.96 | 0.00083 |
| Kcnh7 | -0.392 | ± | 0.210 | 5633.66 | ± | 2341.78 | 3433.18 | ± | 1345.08 | 0.15896 |
| Kctd16 | -0.356 | ± | 0.227 | 818.00 | ± | 287.04 | 484.31 | ± | 174.41 | 0.25641 |
| Kcnj10 | -0.279 | ± | 0.287 | 969.12 | ± | 606.75 | 601.12 | ± | 237.45 | 0.53004 |
| Kcnd1 | -0.261 | ± | 0.180 | 12102.83 | ± | 5958.14 | 7913.03 | ± | 3189.65 | 0.30406 |
| Kctd3 | -0.245 | ± | 0.151 | 1533.61 | ± | 364.46 | 1059.99 | ± | 390.05 | 0.23596 |
| Kcnt1 | -0.225 | ± | 0.199 | 4232.49 | ± | 1910.75 | 2849.31 | ± | 963.93 | 0.44909 |
| Kcng2 | -0.219 | ± | 0.521 | 149.68 | ± | 100.91 | 92.89 | ± | 69.72 | 0.81842 |
| Kcnip4 | -0.207 | ± | 0.196 | 26629.70 | ± | 10076.87 | 18046.19 | ± | 4068.09 | 0.48541 |
| Kcnn1 | -0.196 | ± | 0.317 | 1410.06 | ± | 1105.68 | 916.66 | ± | 211.72 | 0.71883 |
| Kcnq2 | -0.172 | ± | 0.173 | 2328.74 | ± | 470.59 | 1657.66 | ± | 775.46 | 0.51721 |
| Kctd9 | -0.155 | ± | 0.098 | 4385.26 | ± | 293.00 | 3062.40 | ± | 875.79 | 0.25197 |
| Kcnk18 | -0.119 | ± | 0.339 | 981.70 | ± | 416.59 | 745.32 | ± | 250.97 | 0.85282 |
| Kcnn4 | -0.079 | ± | 0.430 | 215.35 | ± | 157.33 | 163.71 | ± | 37.78 | 0.92819 |
| Kctd5 | 0.023 | ± | 0.174 | 2432.67 | ± | 1003.15 | 2060.64 | ± | 723.53 | 0.94876 |
| Kctd1 | 0.027 | ± | 0.273 | 527.96 | ± | 241.39 | 396.10 | ± | 68.94 | 0.964 |
| Kctd21 | 0.081 | ± | 0.313 | 471.88 | ± | 168.09 | 379.99 | ± | 93.44 | 0.89635 |
| Kcnq3 | 0.090 | ± | 0.556 | 336.44 | ± | 363.38 | 201.50 | ± | 13.58 | 0.93727 |
| Kcnj9 | 0.091 | ± | 1.105 | 27.05 | ± | 32.70 | 26.66 | ± | 23.09 | 0.96998 |
| Kcnf1 | 0.131 | ± | 0.652 | 171.46 | ± | 152.91 | 139.35 | ± | 53.79 | 0.92189 |
| Kcnh8 | 0.168 | ± | 0.520 | 235.62 | ± | 170.11 | 218.89 | ± | 129.62 | 0.86493 |
| Kcnc2 | 0.350 | ± | 0.180 | 1671.47 | ± | 434.51 | 1632.99 | ± | 431.78 | 0.13962 |
| Kcnj11 | 0.403 | ± | 0.917 | 21.26 | ± | 11.76 | 23.33 | ± | 14.91 | 0.80882 |
| Kcnmb1 | 0.459 | ± | 0.179 | 42040.63 | ± | 7178.59 | 47040.66 | ± | 14029.19 | 0.03813 |
| Kcng4 | 0.621 | ± | 0.916 | 35.33 | ± | 32.83 | 43.35 | ± | 22.54 | 0.68596 |
| Kcnn3 | 1.157 | ± | 0.880 | 157.82 | ± | 116.04 | 335.24 | ± | 207.58 | 0.35979 |
| Kcnk12 | 1.223 | ± | 1.017 | 3.36 | ± | 1.89 | 5.74 | ± | 4.12 | 0.4129 |
| Kcnk13 | 1.447 | ± | 0.605 | 588.45 | ± | 450.64 | 1292.72 | ± | 475.35 | 0.05705 |
| Kcng3 | 2.009 | ± | 0.950 | 21.26 | ± | 40.17 | 63.98 | ± | 39.93 | 0.10107 |

| **Calcium channels** |  |  |  |  |  |  |  |  |  |  |
| --- | --- | --- | --- | --- | --- | --- | --- | --- | --- | --- |
| MGI symbol | log2 fold change ± SEM | | | Control average counts ± SD | | | Nav-Tsc2 average counts ± SD | | | padj |
| Cacng3 | -3.309 | ± | 0.579 | 692.53 | ± | 125.62 | 63.77 | ± | 72.39 | 1.84E-07 |
| Cacng4 | -2.320 | ± | 0.630 | 192.62 | ± | 44.87 | 29.06 | ± | 23.47 | 0.00151 |
| Cacna1e | -2.246 | ± | 0.553 | 861.03 | ± | 248.07 | 169.93 | ± | 121.49 | 0.00039 |
| Cacnb4 | -1.048 | ± | 0.193 | 8711.76 | ± | 2754.31 | 3477.31 | ± | 1305.89 | 9.00E-07 |
| Cacna1h | -0.932 | ± | 0.338 | 433.90 | ± | 38.81 | 188.88 | ± | 118.86 | 0.02394 |
| Cacna2d2 | -0.627 | ± | 0.221 | 1364.03 | ± | 415.85 | 699.35 | ± | 377.76 | 0.01991 |
| Cacna1c | -0.520 | ± | 0.124 | 4713.93 | ± | 523.82 | 2672.11 | ± | 1075.06 | 0.00022 |
| Cacna2d1 | 0.634 | ± | 0.127 | 22653.70 | ± | 5896.00 | 27661.94 | ± | 9243.52 | 7.66E-06 |
| Cacna1i | 2.360 | ± | 0.887 | 33.20 | ± | 47.29 | 145.64 | ± | 90.58 | 0.03033 |
| Cacnb2 | -1.094 | ± | 0.491 | 284.74 | ± | 146.46 | 99.86 | ± | 31.05 | 0.08012 |
| Cacng5 | -0.572 | ± | 0.457 | 513.88 | ± | 262.34 | 285.45 | ± | 142.36 | 0.38803 |
| Cacna1d | -0.348 | ± | 0.415 | 252.36 | ± | 76.33 | 168.98 | ± | 46.52 | 0.59992 |
| Cacnb3 | -0.096 | ± | 0.107 | 5173.92 | ± | 1432.27 | 3879.53 | ± | 1520.45 | 0.56952 |
| Cacna2d3 | -0.069 | ± | 0.217 | 1079.54 | ± | 265.13 | 805.64 | ± | 327.11 | 0.86849 |
| Cacna1b | -0.034 | ± | 0.089 | 6597.43 | ± | 886.33 | 5126.57 | ± | 1825.23 | 0.83633 |
| Cacnb1 | 0.018 | ± | 0.247 | 884.26 | ± | 130.11 | 743.53 | ± | 440.72 | 0.9738 |
| Cacng2 | 0.127 | ± | 0.311 | 614.39 | ± | 392.96 | 552.74 | ± | 167.32 | 0.82487 |
| Cacna1a | 0.396 | ± | 0.160 | 1046.83 | ± | 239.15 | 1067.88 | ± | 305.04 | 0.04728 |
| Cacng8 | 0.510 | ± | 0.976 | 6.22 | ± | 4.61 | 8.36 | ± | 6.97 | 0.76658 |
| Cacng7 | 0.697 | ± | 0.383 | 29.81 | ± | 8.53 | 37.93 | ± | 14.26 | 0.17243 |

| **Chloride channels** |  |  |  |  |  |  |  |  |  |  |
| --- | --- | --- | --- | --- | --- | --- | --- | --- | --- | --- |
| MGI symbol | log2 fold change ± SEM | | | Control average counts ± SD | | | Nav-Tsc2 average counts ± SD | | | padj |
| Ttyh1 | -0.625 | ± | 0.179 | 1920.30 | ± | 516.59 | 1002.27 | ± | 276.45 | 0.00285 |
| Ttyh2 | 0.515 | ± | 0.163 | 1450.56 | ± | 380.96 | 1641.80 | ± | 684.35 | 0.00779 |
| Clcn5 | 1.574 | ± | 0.152 | 2007.86 | ± | 454.09 | 4519.60 | ± | 1389.88 | 4.92E-23 |
| Clcn2 | -0.752 | ± | 0.517 | 156.70 | ± | 77.75 | 82.03 | ± | 66.05 | 0.30039 |
| Clcn3 | -0.152 | ± | 0.066 | 11735.15 | ± | 864.12 | 8480.58 | ± | 2559.53 | 0.07058 |
| Clcn4 | -0.015 | ± | 0.090 | 10027.95 | ± | 1696.60 | 7823.11 | ± | 2651.20 | 0.9347 |
| Ttyh3 | 0.010 | ± | 0.183 | 736.31 | ± | 115.63 | 626.58 | ± | 311.66 | 0.9792 |
| Clcc1 | 0.036 | ± | 0.210 | 1544.07 | ± | 303.37 | 1233.17 | ± | 218.13 | 0.93411 |
| Clcn6 | 0.302 | ± | 0.109 | 3168.14 | ± | 663.02 | 3141.76 | ± | 1134.59 | 0.02288 |
| Clcn7 | 0.483 | ± | 0.169 | 1548.14 | ± | 327.79 | 1801.26 | ± | 654.99 | 0.01856 |
| Ano1 | 2.087 | ± | 1.544 | 8.49 | ± | 11.13 | 9.78 | ± | 7.81 | 0.34299 |

| **Trp channels** |  |  |  |  |  |  |  |  |  |  |
| --- | --- | --- | --- | --- | --- | --- | --- | --- | --- | --- |
| MGI symbol | log2 fold change ± SEM | | | Control average counts ± SD | | | Nav-Tsc2 average counts ± SD | | | padj |
| Trpc7 | -2.775 | ± | 0.860 | 331.69 | ± | 236.33 | 44.32 | ± | 32.29 | 0.00658 |
| Trpm2 | -1.742 | ± | 0.215 | 1226.91 | ± | 264.99 | 284.80 | ± | 120.08 | 2.71E-14 |
| Trpm6 | -1.559 | ± | 0.381 | 178.07 | ± | 55.77 | 45.07 | ± | 6.09 | 0.00035 |
| Trpc6 | -0.897 | ± | 0.341 | 1507.88 | ± | 756.34 | 657.06 | ± | 271.01 | 0.03281 |
| Trpv2 | 0.748 | ± | 0.125 | 6146.63 | ± | 1454.21 | 8433.98 | ± | 2870.24 | 3.87E-08 |
| Trpv4 | 4.670 | ± | 1.801 | 0.67 | ± | 0.77 | 16.73 | ± | 16.20 | 0.03571 |
| Trpm8 | -0.883 | ± | 0.731 | 737.79 | ± | 697.41 | 310.71 | ± | 247.96 | 0.41016 |
| Trpc5 | -0.674 | ± | 1.477 | 27.04 | ± | 33.43 | 15.17 | ± | 21.39 | 0.79998 |
| Trpv1 | -0.550 | ± | 0.244 | 14867.04 | ± | 5074.95 | 7949.72 | ± | 3611.81 | 0.07639 |
| Trpa1 | -0.499 | ± | 0.228 | 4399.03 | ± | 1456.45 | 2514.27 | ± | 962.42 | 0.08748 |
| Trpc4 | -0.361 | ± | 0.326 | 754.51 | ± | 414.30 | 457.78 | ± | 168.36 | 0.45942 |
| Trpm3 | -0.292 | ± | 0.263 | 795.06 | ± | 356.03 | 533.54 | ± | 220.09 | 0.45893 |
| Trpc1 | -0.003 | ± | 0.157 | 1421.45 | ± | 343.80 | 1095.38 | ± | 384.27 | 0.99261 |
| Trpm4 | 0.165 | ± | 0.205 | 1055.19 | ± | 162.48 | 965.79 | ± | 338.16 | 0.61905 |
| Trpm7 | 0.237 | ± | 0.158 | 1844.70 | ± | 214.38 | 1813.56 | ± | 694.05 | 0.27884 |
| Trpc3 | 0.715 | ± | 0.356 | 5731.67 | ± | 4590.81 | 7531.04 | ± | 2640.53 | 0.12443 |

| **Ligand-gated ion channels** |  |  |  |  |  |  |  |  |  |  |
| --- | --- | --- | --- | --- | --- | --- | --- | --- | --- | --- |
| MGI symbol | log2 fold change ± SEM | | | Control average counts ± SD | | | Nav-Tsc2 average counts ± SD | | | padj |
| Htr3a | -4.302 | ± | 0.857 | 20318.18 | ± | 10525.75 | 1047.35 | ± | 865.90 | 6.42E-06 |
| Chrna7 | -4.199 | ± | 0.586 | 251.47 | ± | 172.10 | 12.63 | ± | 6.35 | 2.49E-11 |
| Grin2d | -4.120 | ± | 0.811 | 106.52 | ± | 58.50 | 4.72 | ± | 4.40 | 4.86E-06 |
| Chrnb4 | -3.957 | ± | 1.114 | 2192.30 | ± | 962.39 | 127.35 | ± | 84.75 | 0.00237 |
| Htr3b | -2.873 | ± | 0.480 | 2477.46 | ± | 1439.22 | 333.85 | ± | 324.91 | 4.01E-08 |
| P2rx6 | -2.385 | ± | 0.725 | 553.21 | ± | 189.09 | 57.19 | ± | 28.57 | 0.00546 |
| Chrna3 | -2.218 | ± | 0.903 | 2042.36 | ± | 1021.91 | 371.48 | ± | 329.78 | 0.04926 |
| P2rx2 | -1.405 | ± | 0.551 | 547.32 | ± | 311.37 | 160.31 | ± | 82.27 | 0.03965 |
| Gria4 | -1.175 | ± | 0.217 | 2340.68 | ± | 729.07 | 886.40 | ± | 388.06 | 9.69E-07 |
| Grik4 | -1.032 | ± | 0.154 | 1613.11 | ± | 412.63 | 620.72 | ± | 159.03 | 5.02E-10 |
| Hcn1 | -0.877 | ± | 0.163 | 7982.93 | ± | 2031.02 | 3543.87 | ± | 1466.55 | 1.12E-06 |
| Gabrg2 | -0.735 | ± | 0.104 | 7450.77 | ± | 1358.97 | 3545.27 | ± | 1090.03 | 4.52E-11 |
| P2rx4 | 0.780 | ± | 0.203 | 1600.08 | ± | 495.59 | 2146.99 | ± | 837.84 | 0.00084 |
| Gabra3 | 0.856 | ± | 0.315 | 739.61 | ± | 167.99 | 1089.23 | ± | 291.61 | 0.02648 |
| Grin3a | 0.902 | ± | 0.230 | 2205.08 | ± | 893.43 | 2884.64 | ± | 831.47 | 0.00066 |
| Chrna6 | 1.128 | ± | 0.230 | 4379.45 | ± | 697.41 | 7880.92 | ± | 2873.37 | 1.07E-05 |
| Hcn3 | 1.262 | ± | 0.265 | 781.68 | ± | 436.45 | 1411.83 | ± | 535.11 | 2.09E-05 |
| Grid1 | 1.527 | ± | 0.295 | 184.39 | ± | 124.65 | 411.35 | ± | 177.15 | 3.14E-06 |
| Grid2 | 1.561 | ± | 0.404 | 169.24 | ± | 120.90 | 437.04 | ± | 177.44 | 0.0008 |
| Gabrb1 | 1.865 | ± | 0.340 | 308.03 | ± | 92.44 | 945.34 | ± | 435.91 | 6.21E-07 |
| Gabrd | -1.791 | ± | 1.287 | 68.33 | ± | 57.11 | 18.49 | ± | 35.12 | 0.32661 |
| Gabra5 | -1.036 | ± | 0.743 | 175.19 | ± | 154.43 | 78.58 | ± | 50.27 | 0.32496 |
| Chrnb3 | -0.824 | ± | 0.636 | 730.66 | ± | 427.53 | 390.60 | ± | 283.77 | 0.36916 |
| Gria3 | -0.479 | ± | 0.325 | 849.78 | ± | 375.00 | 515.36 | ± | 299.71 | 0.29209 |
| Gria2 | -0.135 | ± | 0.123 | 2182.14 | ± | 235.28 | 1616.48 | ± | 619.52 | 0.46254 |
| Gabra2 | -0.077 | ± | 0.128 | 16192.51 | ± | 304.35 | 12197.69 | ± | 4409.42 | 0.72873 |
| Gabra1 | 0.079 | ± | 0.400 | 1661.49 | ± | 1405.10 | 1212.68 | ± | 360.24 | 0.92318 |
| Grin1 | 0.087 | ± | 0.236 | 389.79 | ± | 61.53 | 348.46 | ± | 177.83 | 0.84363 |
| Gabrg3 | 0.147 | ± | 0.765 | 115.51 | ± | 75.37 | 106.75 | ± | 78.77 | 0.9254 |
| Glrb | 0.190 | ± | 0.126 | 4212.50 | ± | 669.74 | 3809.25 | ± | 1415.36 | 0.27791 |
| Grik3 | 0.198 | ± | 0.642 | 203.80 | ± | 168.38 | 212.61 | ± | 150.26 | 0.8711 |
| Grik1 | 0.250 | ± | 0.512 | 15579.78 | ± | 13847.52 | 14590.53 | ± | 3707.89 | 0.78408 |
| Gabrb3 | 0.256 | ± | 0.188 | 2043.38 | ± | 545.88 | 1864.16 | ± | 581.93 | 0.33616 |
| Grik5 | 0.305 | ± | 0.272 | 210.90 | ± | 85.81 | 214.57 | ± | 96.50 | 0.45379 |
| Gria1 | 0.318 | ± | 0.322 | 1958.98 | ± | 944.45 | 1903.17 | ± | 755.22 | 0.52151 |
| Gabbr2 | 0.347 | ± | 0.115 | 5624.38 | ± | 1502.69 | 5569.59 | ± | 1825.69 | 0.01157 |
| Chrnb2 | 0.380 | ± | 0.138 | 1988.19 | ± | 700.13 | 1943.54 | ± | 445.29 | 0.02415 |
| Gabbr1 | 0.407 | ± | 0.129 | 7206.82 | ± | 2143.18 | 7653.74 | ± | 2791.91 | 0.00781 |
| Grina | 0.421 | ± | 0.114 | 28149.39 | ± | 8131.09 | 29321.56 | ± | 11427.01 | 0.00151 |
| P2rx3 | 0.468 | ± | 0.195 | 14233.33 | ± | 6275.58 | 15333.29 | ± | 3837.39 | 0.0559 |
| Hcn2 | 0.529 | ± | 0.483 | 67.25 | ± | 66.19 | 43.59 | ± | 31.74 | 0.46639 |
| P2rx7 | 0.630 | ± | 0.670 | 99.42 | ± | 88.29 | 106.15 | ± | 62.24 | 0.54701 |
| Hcn4 | 0.724 | ± | 0.881 | 13.17 | ± | 13.01 | 15.92 | ± | 6.08 | 0.60959 |
| Grik2 | 0.766 | ± | 0.420 | 278.12 | ± | 87.52 | 338.63 | ± | 80.29 | 0.17068 |
| P2rx5 | 0.778 | ± | 0.493 | 134.97 | ± | 46.64 | 169.31 | ± | 78.08 | 0.25139 |
| Gabrg1 | 0.944 | ± | 0.839 | 354.93 | ± | 289.31 | 502.28 | ± | 354.25 | 0.45111 |
| Chrna4 | 1.037 | ± | 0.627 | 292.53 | ± | 194.83 | 476.66 | ± | 251.08 | 0.22407 |

| **G protein-coupled receptors** |  |  |  |  |  |  |  |  |  |  |
| --- | --- | --- | --- | --- | --- | --- | --- | --- | --- | --- |
| MGI symbol | log2 fold change ± SEM | | | Control average counts ± SD | | | Nav-Tsc2 average counts ± SD | | | padj |
| Ptafr | -7.177 | ± | 0.737 | 1857.76 | ± | 1277.50 | 10.10 | ± | 6.18 | 2.27E-20 |
| Mrgpra3 | -7.033 | ± | 1.255 | 1782.45 | ± | 2134.45 | 4.71 | ± | 5.58 | 3.37E-07 |
| Htr1a | -4.571 | ± | 0.746 | 1125.64 | ± | 478.10 | 39.61 | ± | 24.68 | 1.85E-08 |
| Chrm2 | -4.541 | ± | 1.173 | 705.31 | ± | 75.46 | 28.46 | ± | 43.32 | 0.00078 |
| Prokr2 | -4.061 | ± | 0.667 | 3826.68 | ± | 2970.39 | 196.18 | ± | 95.27 | 2.35E-08 |
| Mrgpra1 | -3.704 | ± | 0.820 | 152.93 | ± | 129.42 | 10.62 | ± | 15.03 | 6.10E-05 |
| Galr1 | -3.346 | ± | 1.267 | 438.93 | ± | 330.40 | 38.81 | ± | 53.22 | 0.03186 |
| Grm8 | -3.325 | ± | 0.631 | 773.89 | ± | 444.52 | 58.08 | ± | 26.49 | 1.90E-06 |
| Npy2r | -3.244 | ± | 0.324 | 5566.80 | ± | 1494.70 | 406.08 | ± | 98.08 | 1.81E-21 |
| Ptgir | -2.875 | ± | 0.429 | 10149.10 | ± | 4141.87 | 1257.11 | ± | 511.16 | 5.79E-10 |
| Htr2a | -2.804 | ± | 0.419 | 268.81 | ± | 119.84 | 30.98 | ± | 6.84 | 5.89E-10 |
| Hcrtr1 | -2.529 | ± | 0.842 | 716.72 | ± | 260.30 | 105.50 | ± | 137.78 | 0.01247 |
| Ptgfr | -2.464 | ± | 0.954 | 601.45 | ± | 560.40 | 52.48 | ± | 40.15 | 0.03646 |
| Adgra1 | -2.318 | ± | 0.256 | 791.77 | ± | 266.62 | 120.38 | ± | 37.96 | 1.03E-17 |
| P2ry2 | -1.722 | ± | 0.439 | 434.24 | ± | 114.78 | 116.02 | ± | 70.24 | 0.00066 |
| Gpr37 | -1.699 | ± | 0.274 | 1794.88 | ± | 493.27 | 493.33 | ± | 247.84 | 1.26E-08 |
| Htr1f | -1.635 | ± | 0.468 | 1425.84 | ± | 642.16 | 304.31 | ± | 113.95 | 0.00287 |
| Htr1d | -1.629 | ± | 0.537 | 989.71 | ± | 634.99 | 196.15 | ± | 57.96 | 0.01152 |
| Ptger1 | -1.572 | ± | 0.399 | 1125.11 | ± | 625.14 | 309.79 | ± | 265.37 | 0.00061 |
| Gpr160 | -1.534 | ± | 0.452 | 640.63 | ± | 309.94 | 162.94 | ± | 16.21 | 0.00393 |
| Cysltr2 | -1.436 | ± | 0.520 | 437.28 | ± | 245.26 | 111.83 | ± | 27.62 | 0.0238 |
| Adgrl1 | -1.093 | ± | 0.179 | 5840.07 | ± | 1190.07 | 2244.80 | ± | 859.81 | 1.94E-08 |
| Gpr149 | -0.837 | ± | 0.282 | 2506.05 | ± | 1077.96 | 1252.31 | ± | 785.47 | 0.01385 |
| Celsr2 | -0.799 | ± | 0.197 | 1456.44 | ± | 314.78 | 694.06 | ± | 239.95 | 0.0004 |
| Adgrl3 | -0.740 | ± | 0.155 | 1670.02 | ± | 483.48 | 786.03 | ± | 289.60 | 1.88E-05 |
| F2rl2 | -0.538 | ± | 0.126 | 30728.43 | ± | 3425.05 | 16497.16 | ± | 5109.75 | 0.00016 |
| Mrgprd | 1.613 | ± | 0.573 | 11070.52 | ± | 10670.22 | 26817.07 | ± | 6593.87 | 0.02076 |
| Adgrl4 | 3.965 | ± | 0.913 | 17.56 | ± | 25.20 | 239.02 | ± | 115.12 | 0.00013 |
| Adgrd1 | 4.017 | ± | 0.367 | 137.03 | ± | 64.07 | 1391.97 | ± | 293.04 | 1.18E-25 |
| Chrm3 | 7.171 | ± | 0.843 | 0.00 | ± | 0.00 | 87.69 | ± | 46.10 | 1.05E-15 |
| Adcyap1r1 | -3.450 | ± | 2.635 | 16.10 | ± | 32.21 | 1.13 | ± | 1.65 | 0.36233 |
| Mrgpra9 | -1.993 | ± | 1.155 | 41.37 | ± | 38.91 | 10.14 | ± | 9.21 | 0.20008 |
| C130060K24Rik | -1.808 | ± | 1.084 | 119.20 | ± | 95.34 | 25.77 | ± | 20.02 | 0.21912 |
| Htr7 | -1.672 | ± | 0.902 | 253.00 | ± | 168.88 | 65.03 | ± | 53.12 | 0.16235 |
| Drd3 | -1.501 | ± | 2.997 | 7.46 | ± | 9.80 | 0.00 | ± | 0.00 | NA |
| Mrgprx1 | -1.053 | ± | 0.782 | 354.02 | ± | 118.35 | 192.42 | ± | 275.65 | 0.34571 |
| Adgrb2 | -0.990 | ± | 1.332 | 15.22 | ± | 16.58 | 8.19 | ± | 5.75 | 0.65224 |
| Adgrb1 | -0.971 | ± | 0.546 | 63.26 | ± | 30.79 | 24.80 | ± | 11.34 | 0.18385 |
| Adgrb1 | -0.971 | ± | 0.546 | 63.26 | ± | 30.79 | 24.80 | ± | 11.34 | 0.18385 |
| Gpr139 | -0.875 | ± | 0.427 | 1257.29 | ± | 582.35 | 447.43 | ± | 197.25 | 0.11545 |
| Gprc5c | -0.745 | ± | 0.316 | 967.08 | ± | 216.12 | 453.13 | ± | 266.94 | 0.06128 |
| Gpr162 | -0.728 | ± | 0.549 | 90.47 | ± | 22.96 | 53.73 | ± | 38.47 | 0.35417 |
| P2ry14 | -0.663 | ± | 0.315 | 487.02 | ± | 205.68 | 253.79 | ± | 79.90 | 0.1036 |
| Adgrg2 | -0.583 | ± | 0.310 | 1648.20 | ± | 700.71 | 774.72 | ± | 285.48 | 0.15528 |
| Adgrg2 | -0.583 | ± | 0.310 | 1648.20 | ± | 700.71 | 774.72 | ± | 285.48 | 0.15528 |
| P2ry1 | -0.564 | ± | 0.409 | 1577.11 | ± | 744.05 | 943.41 | ± | 747.23 | 0.33218 |
| Sstr2 | -0.501 | ± | 1.042 | 915.23 | ± | 927.30 | 617.37 | ± | 832.35 | 0.78733 |
| Adgre5 | -0.402 | ± | 0.649 | 145.02 | ± | 51.11 | 89.88 | ± | 84.71 | 0.71762 |
| Htr5b | -0.364 | ± | 0.516 | 893.56 | ± | 342.47 | 543.23 | ± | 229.93 | 0.67134 |
| Celsr1 | -0.354 | ± | 0.523 | 126.93 | ± | 128.10 | 77.89 | ± | 53.41 | 0.68666 |
| Pth1r | -0.322 | ± | 0.347 | 694.29 | ± | 488.43 | 410.59 | ± | 169.37 | 0.55354 |
| Gpr35 | -0.279 | ± | 0.368 | 2421.40 | ± | 757.74 | 1534.44 | ± | 575.90 | 0.6446 |
| Celsr3 | -0.225 | ± | 0.265 | 851.21 | ± | 304.30 | 595.98 | ± | 259.21 | 0.59579 |
| Gpr3 | -0.139 | ± | 0.695 | 81.69 | ± | 66.35 | 55.94 | ± | 58.08 | 0.92272 |
| Adgra2 | -0.116 | ± | 0.475 | 70.09 | ± | 37.32 | 51.64 | ± | 20.72 | 0.9032 |
| Gprc5b | -0.089 | ± | 0.598 | 149.04 | ± | 84.66 | 118.54 | ± | 33.97 | 0.94257 |
| Adgrb3 | -0.083 | ± | 0.272 | 1047.04 | ± | 562.71 | 825.33 | ± | 322.64 | 0.87304 |
| Grpr | 0.078 | ± | 1.152 | 45.25 | ± | 21.29 | 47.54 | ± | 44.81 | 0.97521 |
| Lpar3 | 0.085 | ± | 0.616 | 3305.54 | ± | 2892.65 | 2520.81 | ± | 862.19 | 0.94666 |
| Adgrl2 | 0.151 | ± | 0.375 | 477.27 | ± | 187.90 | 425.65 | ± | 240.89 | 0.82754 |
| Adgrl2 | 0.151 | ± | 0.375 | 477.27 | ± | 187.90 | 425.65 | ± | 240.89 | 0.82754 |
| Agtr1a | 0.158 | ± | 0.691 | 1509.92 | ± | 1284.51 | 1213.73 | ± | 414.20 | 0.91002 |
| Adgrg6 | 0.195 | ± | 0.310 | 414.04 | ± | 77.66 | 360.72 | ± | 126.07 | 0.71362 |
| Htr4 | 0.215 | ± | 0.449 | 642.89 | ± | 517.72 | 570.97 | ± | 166.77 | 0.78806 |
| Ptger3 | 0.237 | ± | 0.359 | 657.82 | ± | 215.12 | 653.51 | ± | 165.93 | 0.69575 |
| Ptgdr | 0.330 | ± | 0.313 | 2746.74 | ± | 1699.36 | 2677.85 | ± | 724.97 | 0.48751 |
| Adgrg1 | 0.378 | ± | 0.137 | 7845.34 | ± | 2124.77 | 7861.49 | ± | 2542.24 | 0.02418 |
| Hcrtr2 | 0.430 | ± | 0.634 | 619.35 | ± | 284.50 | 629.89 | ± | 382.76 | 0.68586 |
| Ednra | 0.538 | ± | 0.663 | 182.36 | ± | 58.04 | 193.70 | ± | 62.87 | 0.61474 |
| Lpar5 | 0.569 | ± | 0.682 | 69.28 | ± | 58.04 | 76.36 | ± | 62.94 | 0.60203 |
| Oprl1 | 0.665 | ± | 0.323 | 1030.14 | ± | 306.62 | 1419.97 | ± | 498.59 | 0.11316 |
| Sstr4 | 0.747 | ± | 2.647 | 24.25 | ± | 41.97 | 4.13 | ± | 8.26 | NA |
| Adgrf5 | 0.846 | ± | 0.695 | 374.43 | ± | 328.28 | 621.55 | ± | 429.73 | 0.40539 |
| Adgrf5 | 0.846 | ± | 0.695 | 374.43 | ± | 328.28 | 621.55 | ± | 429.73 | 0.40539 |
| Adgra3 | 0.872 | ± | 0.422 | 585.03 | ± | 308.68 | 728.38 | ± | 312.96 | 0.1113 |
| Mc4r | 1.188 | ± | 1.567 | 15.77 | ± | 31.55 | 15.94 | ± | 11.71 | 0.64444 |
| Adgrf4 | 1.386 | ± | 1.385 | 9.96 | ± | 13.65 | 15.72 | ± | 3.93 | 0.51431 |
| Adra1d | 1.551 | ± | 1.519 | 2.15 | ± | 1.46 | 8.10 | ± | 6.93 | 0.5043 |
| Adgrv1 | 1.583 | ± | 1.072 | 13.56 | ± | 15.02 | 38.65 | ± | 41.13 | 0.29067 |
| Adgre1 | 2.589 | ± | 2.949 | 12.82 | ± | 25.63 | 6.07 | ± | 8.24 | NA |
| Adgrg3 | 3.548 | ± | 2.382 | 0.99 | ± | 1.26 | 9.52 | ± | 18.35 | 0.28543 |

| **Transcription factors** |  |  |  |  |  |  |  |  |  |  |
| --- | --- | --- | --- | --- | --- | --- | --- | --- | --- | --- |
| MGI symbol | log2 fold change ± SEM | | | Control average counts ± SD | | | Nav-Tsc2 average counts ± SD | | | padj |
| Bcl11a | -2.953 | ± | 0.554 | 674.10 | ± | 359.67 | 85.85 | ± | 90.13 | 1.44E-06 |
| Etv1 | -2.894 | ± | 0.381 | 2506.69 | ± | 1234.21 | 261.55 | ± | 149.83 | 1.31E-12 |
| Pou4f3 | -2.227 | ± | 0.450 | 4514.91 | ± | 938.73 | 799.17 | ± | 191.43 | 9.19E-06 |
| Dach1 | -2.197 | ± | 0.821 | 178.48 | ± | 91.19 | 32.76 | ± | 21.09 | 0.0293 |
| Etv5 | -2.087 | ± | 0.224 | 2414.18 | ± | 294.60 | 503.77 | ± | 301.07 | 9.90E-19 |
| Zcchc12 | -1.898 | ± | 0.383 | 5029.41 | ± | 2664.77 | 1050.76 | ± | 605.33 | 8.68E-06 |
| Cux2 | -1.823 | ± | 0.467 | 2678.42 | ± | 1764.65 | 688.81 | ± | 354.60 | 0.00069 |
| Maf | -1.689 | ± | 0.184 | 1997.37 | ± | 239.06 | 519.67 | ± | 184.00 | 3.54E-18 |
| Lcorl | -1.678 | ± | 0.326 | 3353.37 | ± | 1115.38 | 905.05 | ± | 333.50 | 3.50E-06 |
| Camta1 | -0.939 | ± | 0.064 | 40373.46 | ± | 3587.99 | 16672.59 | ± | 4650.31 | 3.33E-45 |
| Prdm8 | -0.823 | ± | 0.239 | 740.79 | ± | 203.06 | 351.97 | ± | 146.81 | 0.00326 |
| Creg1 | 0.846 | ± | 0.290 | 224.31 | ± | 99.30 | 298.94 | ± | 131.34 | 0.01559 |
| Pdlim1 | 0.959 | ± | 0.318 | 989.71 | ± | 761.04 | 1402.33 | ± | 434.78 | 0.01211 |
| Grhl3 | 1.108 | ± | 0.425 | 684.78 | ± | 498.23 | 1146.18 | ± | 527.52 | 0.03437 |
| Lrrfip1 | 1.166 | ± | 0.378 | 458.76 | ± | 239.05 | 766.86 | ± | 244.70 | 0.01001 |
| Elk3 | 1.480 | ± | 0.334 | 277.79 | ± | 187.52 | 549.77 | ± | 136.73 | 8.55E-05 |
| Pde8a | 2.633 | ± | 0.364 | 227.35 | ± | 83.92 | 1130.68 | ± | 409.29 | 1.74E-11 |
| Ppargc1b | 2.988 | ± | 0.302 | 154.19 | ± | 33.64 | 950.64 | ± | 413.94 | 4.85E-21 |
| Esrrb | 5.195 | ± | 1.659 | 0.00 | ± | 0.00 | 14.14 | ± | 10.64 | 0.00867 |
| Meis2 | -0.823 | ± | 0.550 | 281.35 | ± | 95.43 | 99.27 | ± | 14.95 | 0.28232 |
| Hoxb8 | -1.621 | ± | 1.176 | 33.66 | ± | 35.20 | 8.47 | ± | 5.90 | 0.33154 |
| Hoxb9 | -1.291 | ± | 0.837 | 518.91 | ± | 612.83 | 163.39 | ± | 98.57 | 0.26529 |
| Fli1 | -1.224 | ± | 0.642 | 319.74 | ± | 239.23 | 97.67 | ± | 41.54 | 0.14779 |
| Zbtb16 | -1.207 | ± | 1.534 | 28.21 | ± | 34.64 | 12.41 | ± | 11.38 | 0.62843 |
| Ets1 | -1.176 | ± | 0.517 | 580.99 | ± | 525.13 | 188.46 | ± | 36.77 | 0.07258 |
| Irf6 | -1.091 | ± | 0.540 | 725.92 | ± | 421.52 | 302.84 | ± | 186.85 | 0.12079 |
| Satb1 | -0.685 | ± | 0.299 | 1143.12 | ± | 423.58 | 524.21 | ± | 128.83 | 0.07003 |
| Shox2 | -0.434 | ± | 1.298 | 95.14 | ± | 110.70 | 23.98 | ± | 23.29 | 0.86006 |
| Isl2 | -0.417 | ± | 0.155 | 6534.63 | ± | 836.27 | 4084.11 | ± | 1400.90 | 0.02795 |
| Pknox2 | -0.409 | ± | 0.193 | 3189.97 | ± | 1047.24 | 1953.75 | ± | 469.90 | 0.10033 |
| Ppargc1a | -0.317 | ± | 0.227 | 940.02 | ± | 189.73 | 587.50 | ± | 244.43 | 0.32261 |
| Hey2 | -0.240 | ± | 0.287 | 616.21 | ± | 142.66 | 426.57 | ± | 106.21 | 0.60164 |
| Casz1 | -0.192 | ± | 0.217 | 2950.15 | ± | 1399.46 | 2160.98 | ± | 747.28 | 0.57476 |
| Nr1d2 | -0.182 | ± | 0.243 | 564.87 | ± | 111.50 | 380.18 | ± | 64.23 | 0.6481 |
| Klf5 | -0.112 | ± | 0.285 | 1444.20 | ± | 810.34 | 1052.08 | ± | 380.31 | 0.83301 |
| Onecut1 | 0.017 | ± | 1.188 | 7.17 | ± | 7.62 | 4.30 | ± | 1.79 | 0.99411 |
| Creb3l1 | 0.121 | ± | 0.356 | 863.30 | ± | 270.90 | 637.44 | ± | 168.31 | 0.85713 |
| Bnc2 | 0.149 | ± | 0.186 | 1042.47 | ± | 255.18 | 909.65 | ± | 224.61 | 0.61898 |
| Rarg | 0.165 | ± | 0.324 | 294.30 | ± | 216.17 | 244.32 | ± | 60.38 | 0.77236 |
| Myt1 | 0.272 | ± | 0.195 | 3061.90 | ± | 1211.41 | 2895.52 | ± | 895.14 | 0.32606 |
| Elf1 | 0.279 | ± | 0.196 | 1723.32 | ± | 323.63 | 1537.71 | ± | 497.08 | 0.31267 |
| Esrrg | 0.304 | ± | 1.619 | 97.25 | ± | 130.29 | 25.31 | ± | 31.66 | 0.92688 |
| Insm2 | 0.307 | ± | 0.281 | 1275.39 | ± | 878.83 | 1200.02 | ± | 344.61 | 0.46696 |
| Scrt1 | 0.351 | ± | 0.479 | 44.86 | ± | 29.76 | 39.10 | ± | 22.75 | 0.65715 |
| Ebf3 | 0.359 | ± | 0.153 | 2422.70 | ± | 836.42 | 2516.47 | ± | 764.81 | 0.06301 |
| Runx1 | 0.396 | ± | 0.200 | 6491.69 | ± | 2980.06 | 6536.78 | ± | 1428.67 | 0.13005 |
| Klf12 | 0.403 | ± | 1.345 | 12.22 | ± | 12.99 | 7.10 | ± | 6.45 | 0.87581 |
| Hoxc8 | 0.439 | ± | 0.265 | 237.41 | ± | 58.10 | 236.92 | ± | 65.28 | 0.22305 |
| Smad9 | 0.454 | ± | 0.184 | 948.39 | ± | 365.09 | 1012.80 | ± | 383.41 | 0.04773 |
| Lbh | 0.572 | ± | 0.300 | 1076.43 | ± | 792.70 | 1175.14 | ± | 321.52 | 0.14859 |
| Egr2 | 0.658 | ± | 0.897 | 109.95 | ± | 132.84 | 109.49 | ± | 55.20 | 0.65715 |
| Pgr | 0.713 | ± | 0.681 | 154.24 | ± | 134.48 | 207.26 | ± | 99.07 | 0.49022 |
| Zfp423 | 0.850 | ± | 0.399 | 318.33 | ± | 213.34 | 410.40 | ± | 135.72 | 0.09852 |
| Npas2 | 0.876 | ± | 0.482 | 92.94 | ± | 75.37 | 130.63 | ± | 72.75 | 0.17346 |
| Zfp57 | 1.356 | ± | 0.296 | 521.68 | ± | 236.19 | 1151.58 | ± | 527.14 | 4.55E-05 |
| Vdr | 2.279 | ± | 1.322 | 13.09 | ± | 26.18 | 43.88 | ± | 29.47 | 0.20086 |
| Runx3 | 2.433 | ± | 1.910 | 1.55 | ± | 1.18 | 15.78 | ± | 18.88 | 0.3782 |

| **IB4-positive enriched** |  |  |  |  |  |  |  |  |  |  |
| --- | --- | --- | --- | --- | --- | --- | --- | --- | --- | --- |
| MGI symbol | log2 fold change ± SEM | | | Control average counts ± SD | | | Nav-Tsc2 average counts ± SD | | | padj |
| Ms4a3 | -3.210 | ± | 0.767 | 1049.85 | ± | 939.53 | 87.66 | ± | 83.73 | 0.00024 |
| Mab21l1 | -2.056 | ± | 0.580 | 1443.58 | ± | 1326.90 | 239.31 | ± | 43.27 | 0.0024 |
| Gpc3 | -1.600 | ± | 0.510 | 820.32 | ± | 607.57 | 195.42 | ± | 82.33 | 0.00847 |
| Rab27b | -0.922 | ± | 0.217 | 8542.73 | ± | 4099.47 | 3551.71 | ± | 1047.38 | 0.00019 |
| Trpc6 | -0.897 | ± | 0.341 | 1507.88 | ± | 756.34 | 657.06 | ± | 271.01 | 0.03281 |
| Cpn1 | -0.786 | ± | 0.313 | 806.77 | ± | 114.54 | 365.36 | ± | 151.35 | 0.04354 |
| Moxd1 | 1.219 | ± | 0.429 | 1554.71 | ± | 1358.05 | 3196.08 | ± | 1479.99 | 0.01928 |
| Ggta1 | 1.398 | ± | 0.351 | 1293.41 | ± | 1081.17 | 2657.64 | ± | 682.57 | 0.00052 |
| Serping1 | 1.561 | ± | 0.386 | 11125.74 | ± | 6706.44 | 25813.67 | ± | 7161.58 | 0.00041 |
| Mrgprd | 1.613 | ± | 0.573 | 11070.52 | ± | 10670.22 | 26817.07 | ± | 6593.87 | 0.02076 |
| Gpnmb | 2.147 | ± | 0.547 | 986.42 | ± | 1042.21 | 3241.30 | ± | 1489.09 | 0.00065 |
| Dnajc5b | 2.223 | ± | 0.692 | 55.53 | ± | 69.63 | 179.11 | ± | 83.24 | 0.00681 |
| Nnat | -1.533 | ± | 0.688 | 235.47 | ± | 183.17 | 58.09 | ± | 38.48 | 0.0803 |
| Hal | -0.990 | ± | 0.634 | 1039.17 | ± | 910.00 | 367.94 | ± | 94.26 | 0.25798 |
| Wnt2b | -1.680 | ± | 1.024 | 58.80 | ± | 52.71 | 12.97 | ± | 16.20 | 0.22849 |
| Lypd1 | -1.615 | ± | 1.603 | 50.84 | ± | 72.91 | 13.08 | ± | 15.03 | 0.51068 |
| A3galt2 | -0.566 | ± | 0.407 | 1430.30 | ± | 1189.24 | 767.02 | ± | 304.39 | 0.32647 |
| 9430021M05Rik | -0.200 | ± | 0.303 | 304.91 | ± | 76.42 | 217.33 | ± | 93.93 | 0.69614 |
| Klk5 | 0.007 | ± | 0.551 | 484.13 | ± | 458.26 | 395.43 | ± | 210.37 | 0.99465 |
| Prkcq | 0.022 | ± | 0.652 | 4019.55 | ± | 3813.99 | 3175.37 | ± | 789.72 | 0.98694 |
| Lpar3 | 0.085 | ± | 0.616 | 3305.54 | ± | 2892.65 | 2520.81 | ± | 862.19 | 0.94666 |
| Cyp4f39 | 0.099 | ± | 0.536 | 325.81 | ± | 196.01 | 282.27 | ± | 159.45 | 0.92835 |
| Agtr1a | 0.158 | ± | 0.691 | 1509.92 | ± | 1284.51 | 1213.73 | ± | 414.20 | 0.91002 |
| Ptprt | 0.196 | ± | 0.199 | 6056.55 | ± | 2669.39 | 5677.84 | ± | 1721.31 | 0.52448 |
| Htr4 | 0.215 | ± | 0.449 | 642.89 | ± | 517.72 | 570.97 | ± | 166.77 | 0.78806 |
| Paqr5 | 0.228 | ± | 0.337 | 8067.54 | ± | 5685.79 | 6918.99 | ± | 1404.84 | 0.68719 |
| Grik1 | 0.250 | ± | 0.512 | 15579.78 | ± | 13847.52 | 14590.53 | ± | 3707.89 | 0.78408 |
| Ptgdr | 0.330 | ± | 0.313 | 2746.74 | ± | 1699.36 | 2677.85 | ± | 724.97 | 0.48751 |
| St6gal2 | 0.426 | ± | 0.398 | 665.24 | ± | 476.34 | 697.81 | ± | 220.22 | 0.47899 |
| Klhl5 | 0.512 | ± | 0.323 | 8707.44 | ± | 6350.66 | 9761.16 | ± | 3366.41 | 0.24889 |
| Tmem79 | 0.540 | ± | 0.295 | 2522.91 | ± | 1204.76 | 2783.98 | ± | 922.00 | 0.16933 |
| Ccdc68 | 0.543 | ± | 0.435 | 4156.79 | ± | 3073.90 | 4650.78 | ± | 1368.65 | 0.39131 |
| Lpar5 | 0.569 | ± | 0.682 | 69.28 | ± | 58.04 | 76.36 | ± | 62.94 | 0.60203 |
| Slc16a12 | 0.700 | ± | 0.296 | 935.49 | ± | 594.03 | 1182.80 | ± | 327.77 | 0.06031 |
| Trpc3 | 0.715 | ± | 0.356 | 5731.67 | ± | 4590.81 | 7531.04 | ± | 2640.53 | 0.12443 |
| Ctxn3 | 0.737 | ± | 0.313 | 25934.59 | ± | 19308.12 | 33270.63 | ± | 7344.83 | 0.06168 |
| Rassf9 | 0.748 | ± | 0.490 | 178.94 | ± | 50.30 | 233.51 | ± | 103.61 | 0.27166 |
| Adgrf5 | 0.846 | ± | 0.695 | 374.43 | ± | 328.28 | 621.55 | ± | 429.73 | 0.40539 |

| **IB4-negative enriched** |  |  |  |  |  |  |  |  |  |  |
| --- | --- | --- | --- | --- | --- | --- | --- | --- | --- | --- |
| MGI symbol | log2 fold change ± SEM | | | Control average counts ± SD | | | Nav-Tsc2 average counts ± SD | | | padj |
| Sst | -8.951 | ± | 0.926 | 4678.26 | ± | 2981.34 | 6.95 | ± | 6.55 | 4.26E-20 |
| Hoxd1 | -6.622 | ± | 0.605 | 758.01 | ± | 179.39 | 5.77 | ± | 6.58 | 1.29E-25 |
| Il31ra | -5.361 | ± | 0.728 | 1427.07 | ± | 879.47 | 26.97 | ± | 27.04 | 6.37E-12 |
| Htr1a | -4.571 | ± | 0.746 | 1125.64 | ± | 478.10 | 39.61 | ± | 24.68 | 1.85E-08 |
| Cgnl1 | -4.491 | ± | 0.673 | 4369.28 | ± | 1143.06 | 201.76 | ± | 166.60 | 6.76E-10 |
| Chp2 | -4.408 | ± | 0.840 | 1489.65 | ± | 642.61 | 72.59 | ± | 56.63 | 2.13E-06 |
| Fam26d | -4.178 | ± | 1.259 | 891.43 | ± | 449.73 | 42.83 | ± | 51.96 | 0.00497 |
| Prokr2 | -4.061 | ± | 0.667 | 3826.68 | ± | 2970.39 | 196.18 | ± | 95.27 | 2.35E-08 |
| H2-M11 | -4.022 | ± | 1.539 | 480.00 | ± | 332.34 | 36.21 | ± | 62.17 | 0.03395 |
| Gng8 | -3.827 | ± | 0.509 | 5777.77 | ± | 2327.25 | 380.24 | ± | 376.33 | 2.13E-12 |
| Fam20a | -3.774 | ± | 0.619 | 828.56 | ± | 337.37 | 59.52 | ± | 36.42 | 2.20E-08 |
| Nppb | -3.453 | ± | 0.871 | 3762.87 | ± | 2592.72 | 185.74 | ± | 147.83 | 0.00056 |
| Stk32c | -3.329 | ± | 0.631 | 258.49 | ± | 73.23 | 20.56 | ± | 12.45 | 1.87E-06 |
| Scgn | -3.284 | ± | 0.861 | 589.63 | ± | 450.59 | 67.34 | ± | 56.62 | 0.00095 |
| Npy2r | -3.244 | ± | 0.324 | 5566.80 | ± | 1494.70 | 406.08 | ± | 98.08 | 1.81E-21 |
| Ptgir | -2.875 | ± | 0.429 | 10149.10 | ± | 4141.87 | 1257.11 | ± | 511.16 | 5.79E-10 |
| S100a16 | -2.797 | ± | 0.526 | 6921.13 | ± | 2868.42 | 902.09 | ± | 849.06 | 1.50E-06 |
| Hgf | -2.743 | ± | 0.484 | 1271.82 | ± | 669.78 | 187.99 | ± | 165.49 | 2.40E-07 |
| Trhde | -2.645 | ± | 0.896 | 360.17 | ± | 175.68 | 50.32 | ± | 56.68 | 0.01436 |
| Trappc3l | -2.556 | ± | 0.427 | 21828.38 | ± | 6421.73 | 3367.23 | ± | 1819.25 | 4.23E-08 |
| Kcnt2 | -2.180 | ± | 0.252 | 2287.87 | ± | 444.11 | 432.68 | ± | 180.79 | 3.41E-16 |
| Prlr | -2.118 | ± | 0.629 | 2046.73 | ± | 1342.77 | 426.53 | ± | 135.95 | 0.00426 |
| Nrp2 | -2.101 | ± | 0.412 | 3950.22 | ± | 2573.51 | 848.60 | ± | 430.94 | 4.42E-06 |
| Serpinb1b | -1.849 | ± | 0.606 | 5101.51 | ± | 2356.11 | 1362.73 | ± | 1380.98 | 0.01088 |
| Ndp | -1.648 | ± | 0.263 | 5074.24 | ± | 1167.33 | 1294.33 | ± | 320.06 | 7.62E-09 |
| Mgll | -1.055 | ± | 0.241 | 6562.76 | ± | 1923.90 | 2707.08 | ± | 1645.21 | 0.00011 |
| Abcg2 | -0.990 | ± | 0.346 | 36711.54 | ± | 12806.88 | 14969.61 | ± | 5812.57 | 0.01836 |
| Ptk2b | -0.879 | ± | 0.320 | 2295.72 | ± | 1382.52 | 1083.74 | ± | 690.51 | 0.02466 |
| Penk | 2.948 | ± | 0.807 | 3434.64 | ± | 3043.68 | 23666.70 | ± | 24877.61 | 0.00169 |
| Bmp15 | -1.429 | ± | 1.733 | 286.25 | ± | 282.75 | 71.09 | ± | 105.11 | 0.60817 |
| Tyrp1 | -1.328 | ± | 0.660 | 470.55 | ± | 49.19 | 167.77 | ± | 75.83 | 0.12306 |
| C130060K24Rik | -1.808 | ± | 1.084 | 119.20 | ± | 95.34 | 25.77 | ± | 20.02 | 0.21912 |
| Loxl4 | -2.667 | ± | 1.209 | 116.14 | ± | 72.45 | 17.36 | ± | 15.11 | 0.084 |
| Naip1 | -2.648 | ± | 1.329 | 126.72 | ± | 77.73 | 20.13 | ± | 33.66 | 0.12717 |
| Mctp1 | -0.863 | ± | 0.369 | 6268.19 | ± | 1907.15 | 2498.88 | ± | 950.09 | 0.06347 |
| Cyp1b1 | -0.631 | ± | 0.415 | 4405.10 | ± | 2287.64 | 2211.04 | ± | 576.37 | 0.27244 |
| Pon3 | -0.305 | ± | 0.641 | 662.05 | ± | 454.37 | 441.62 | ± | 188.35 | 0.78959 |
| Scin | 0.152 | ± | 0.707 | 435.65 | ± | 84.45 | 444.81 | ± | 319.98 | 0.91564 |
| Srd5a2 | 3.584 | ± | 2.959 | 6.40 | ± | 12.80 | 3.86 | ± | 4.47 | NA |
